# Challenges in detecting and predicting adverse drug events via distributed analysis of electronic health record data from German university hospitals

**DOI:** 10.1371/journal.pdig.0000892

**Published:** 2025-06-26

**Authors:** Anna Maria Wermund, Torsten Thalheim, André Medek, Florian Schmidt, Thomas Peschel, Alexander Strübing, Daniel Neumann, André Scherag, Markus Loeffler, Miriam Kesselmeier, Ulrich Jaehde, for the POLAR_MI Consortium

**Affiliations:** 1 Department of Clinical Pharmacy, Institute of Pharmacy, University of Bonn, Bonn, Germany; 2 Interdisciplinary Centre for Bioinformatics, Leipzig University, Leipzig, Germany; 3 Institute for Medical Informatics, Statistics and Epidemiology (IMISE), Leipzig University, Leipzig, Germany; 4 Deutsches Biomasseforschungszentrum gGmbH, Leipzig, Germany; 5 Medical & Scientific Technology Development & Coordination (MWTek), University Hospital Bonn, Bonn, Germany; 6 Institute of Medical Statistics, Computer and Data Sciences (IMSID), Jena University Hospital – Friedrich Schiller University Jena, Jena, Germany; Instituto Politécnico Nacional Escuela Superior de Medicina: Instituto Politecnico Nacional Escuela Superior de Medicina, MEXICO

## Abstract

The Medical Informatics Initiative Germany (MII) aims to facilitate the interoperability and exchange of electronic health record data from all German university hospitals. The MII use case “POLyphamacy, drug interActions and Risks” (POLAR_MI) was designed to retrospectively detect medication-related risks in adult inpatients. As part of POLAR_MI, we aimed to build predictive models for specific adverse events. Here, using the two adverse events gastrointestinal bleeding and drug-related hypoglycaemia as examples, we present our initial investigation to determine whether these adverse events and their associations with potential risk factors can be detected. We applied a two-step distributed analysis approach to electronic health record data from 2018 to 2021. This approach consisted of a local statistical data analysis at ten participating centres, followed by a mixed-effects meta-analysis. For each adverse event, two multivariable logistic regression models were constructed: (1) including only demographics, diagnoses and medications, and (2) extended by laboratory values. As numerically stable estimations of both models were not possible at each centre, we constructed different centre subgroups for meta-analyses. We received prevalence estimates of around 1.2% for GI bleeding and around 3.0% for drug-related hypoglycaemia. Although unavailability of laboratory values was a common reason hindering model estimation, multivariable regression models were obtained for both adverse events from several centres. Regarding our original intention to build predictive models, the median area under the receiver operating characteristic curve was above 0.70 for all multivariable regression models, indicating feasibility. In conclusion, plausible estimates for prevalence and regression modelling odds ratios were received when using a distributed analysis approach on inpatient treatment data from diverse German university hospitals. Our results suggest that the development of predictive models in a distributed setting is possible if the research question is adapted to the infrastructure and the available data.

## Author summary

Undesirable medical occurrences following exposure to a drug are called adverse events. They are a common and often preventable cause of illness in patients. To facilitate their detection and prevention, models can be used to predict the probability of the development or presence of an adverse event. As part of the project “POLypharmacy, drug interActions and Risks” (POLAR_MI) of the Medical Informatics Initiative Germany (MII), we investigated whether two adverse events (gastrointestinal bleeding and drug-related hypoglycaemia) could be detected and their associations with potential risk factors assessed. This was done to test the feasibility of building predictive models within the framework established by the MII using a distributed analysis approach without direct data access for the analyst. We received plausible estimates for the prevalence of the adverse events and risk factors, and observed mainly clinically plausible associations between the adverse events and the related risk factors. One of our major challenges was the unavailability of laboratory values used to define the adverse event drug-related hypoglycaemia and some potential risk factors. Based on our results, we conclude that the development of predictive models is possible if a research question can be defined that fits the IT infrastructure and the available data.

## 1. Introduction

Germany has 36 university hospitals, of which each runs its independent hospital information system (HIS) [1]. An integral part of such HIS are electronic health records (EHR). The cross-institutional exchange and statistical analyses of EHR data from these hospitals would offer an opportunity to gain insights into health risks and subsequently improve decision making in healthcare at local, national, and global levels [2–5]. The realisation requires improvements in local data quality, processing and analysis, as well as the rapid and standardised cross-institutional exchange of EHR data. However, data protection regulations and the use of diverse clinical systems, resulting in a lack of semantic, syntactic and organisational interoperability, impose a challenge to data sharing between different university hospitals [6,7]. At the local level, the integration of various health IT systems (hospital software, laboratory systems, imaging systems, etc.) to work seamlessly with the HIS infrastructure remains challenging. At the cross-institutional level, various HIS infrastructures with different documentation standards complicate data exchange.

Against this background, the Medical Informatics Initiative Germany (MII) has been working since 2018 to establish decentralised data infrastructures at German university hospitals to facilitate better interoperability between systems, thus enabling data sharing of EHR data and subsequent multicentre analyses [8]. For this purpose, data integration centres (DIC) were established. At the DIC, data from EHR are available via the MII Core Data Set (CDS), where HL7 FHIR (Health Level Seven; Fast Health Interoperability Resources; http://hl7.org/fhir/ [9]; “FHIR is the registered trademark of HL7 and is used with the permission of HL7; use of the FHIR trademark does not constitute endorsement of this product by HL7”) is used as interoperable data format [10–12].

Applying the MII processes and methods to data from routine hospital healthcare, the collaborative use case “POLypharmacy, drug interActions and Risks” (POLAR_MI) was designed to retrospectively detect medication-related risks, e.g., adverse events (AE) and adverse drug events (ADE), in hospitalised adults [13]. This objective was the starting point to demonstrate that it is possible to retrieve and use healthcare data within the MII on a large scale. For this purpose, the “POLAR_MI ETL Pipeline”, a tailored distributed analysis approach, was developed to conduct research projects related to the overall aim of POLAR_MI [13].

In our research project within POLAR_MI, we pursued the aim to build models predicting specific inpatient ADE based on information available at hospital admission. Such models can be used to prioritise services such as medication reviews or medication reconciliation for patients at high risk for ADE, thereby facilitating the efficient use of available resources and leading to a greater impact on medication safety [14–17]. Many models predicting ADE have already been developed, often based on prospective studies tailored to this research question, giving the researchers control over the required data and the related documentation quality [17,18]. However, due to the controlled conditions of these studies, they only partly reflect real inpatient care. By contrast, data from EHR, as used in the context of the MII, originate from the documentation of treatment and care. Such data from routine healthcare, of unknown quality and completeness, are increasingly used to complement data from prospective studies for the development of predictive models [19].

Here we present our initial multicentre investigation of whether AE can be detected and their association with potential risk factors assessed (via logistic regression modelling) using a distributed analysis approach. We analysed this feasibility based on two AE (gastrointestinal bleeding, drug-related hypoglycaemia), including an assessment of the impact of integrating laboratory values and considering the chronology of events.

## 2. Materials and methods

### 2.1 The POLAR_MI framework

#### 2.1.1 Study design and setting.

POLAR_MI was a multicentre, retrospective observational study at German university hospitals. The POLAR_MI-wide inclusion criteria were: (1) admission and discharge within the time interval 2018/01/01 and 2021/12/31, (2) inpatient hospital stay and (3) age ≥ 18 years on admission. Additionally, no technical reasons hindering data processing were permitted to be present.

**Trial registration:** POLAR_MI was registered on 27/11/2020 in the “HMA-EMA Catalogues of real-world data sources and studies” (EU PAS number: EUPAS36582).

**Ethics statement:** POLAR_MI received ethics approvals from all participating centres. The primary approval was granted by the Ethics Committee at the Medical Faculty of the University Leipzig (lead ethics committee). The names of the relevant ethics committees and the respective reference numbers are provided in Table 1.

**Table 1:** Ethics committees and the reference numbers of the participating centres. Abbreviation: -, not available.

Participating centre	Ethics Committee		Reference number
	Official German name	Official English name	
Bonn	Ethikkommission an der Medizinischen Fakultät der Rheinischen Friedrich-Wilhelms-Universität Bonn	Ethics Committee at the Medical Faculty of the Rheinische Friedrich-Wilhelms-Universität Bonn	101/21
Erlangen	Ethik-Kommission an der Medizinischen Fakultät der Friedrich-Alexander-Universität Erlangen-Nürnberg	–	368_20 Bc
Freiburg/Breisgau	Ethik-Kommission der Albert-Ludwigs-Universität Freiburg	Ethics Committee of the Albert Ludwig University of Freiburg	20-1267
Gießen	Ethik-Kommission des Fachbereichs Medizin der Justus-Liebig-Universität Gießen	Ethics Committee of the Faculty of Medicine at Justus Liebig University Giessen	adopted the approval granted by the lead ethics committee (Leipzig)
Halle/Saale	Ethik-Kommission der Medizinischen Fakultät der Martin-Luther-Universität Halle-Wittenberg	Ethics Committee at the Medical Faculty of the Martin Luther University Halle-Wittenberg	2021-119
Hamburg	Ethik-Kommission der Ärztekammer Hamburg	–	2020-10251-BO-bet
Heidelberg	Universität Heidelberg Ethikkommission der Me-di-zi-ni-schen Fakultät	Ethics Committee of the Medical Faculty of Heidelberg University	S-240/2021
Jena	Universitätsklinikum Jena Ethik-Kommission	–	2020-1931-Daten
Kiel	Ethik-Kommission der Medizinischen Fakultät der Christian-Albrechts-Universität zu Kiel	Ethics Committee at the Faculty of Medicine of Kiel University	B 280/21
Leipzig (lead)	Ethik-Kommission an der Medizinischen Fakultät der Universität Leipzig	Ethics Committee at the Medical Faculty of the University Leipzig	247/20-ek
München	Ethikkommission der Ludwig-Maximilians Universität München	–	20-0961
Tübingen	Ethik-Kommission an der Medizinischen Fakultät der Eberhard-Karls-Universität und am Universitätsklinikum Tübingen	The Ethics Committee at the Medical Faculty of the Eberhard Karls University and at the University Hospital of Tübingen	712/2020BO2

**Patient consent:** Informed patient consent was not required due to the retrospective, distributed analysis approach in POLAR_MI, where only anonymised data (i.e., summary statistics) not falling under der EU General Data Protection Regulation left the centres.

#### 2.1.2 Data collection and (pre-) processing.

**Fig 1:**
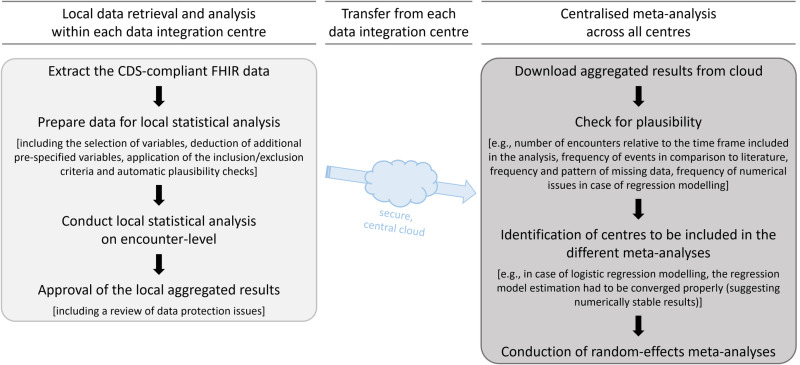
The POLAR_MI ETL Pipeline in a nutshell: A short illustration of the applied two-step distributed analysis approach. For both steps, the sequence of the related tasks (including examples for clarification) is provided. Abbreviations: CDS, core data set; ETL, extract – transform – load.

We applied the POLAR_MI ETL Pipeline, which is a two-step distributed analysis approach (see Fig 1). In the first step, modules (i.e., a composition of several R scripts) for data retrieval, preparation and statistical analysis on encounter-level were executed locally at each DIC on the CDS-compliant FHIR data. In our analysis, an encounter corresponded to an inpatient hospital stay. After approval of the local aggregated results by the DIC, including a review of data protection issues, we pooled the local results in a second step via random-effects meta-analyses, accounting for heterogeneity of the participating centres. For our research project, we received local results from ten centres (Bonn, Erlangen, Freiburg/Breisgau, Gießen, Halle/Saale, Hamburg, Heidelberg, Jena, Kiel, Leipzig). We used R (version 4.2.2) and the R packages *meta* (version 6.0.0) and *metamedian* (version 0.1.5) for the meta-analyses [20–22].

### 2.2 Tailoring the POLAR_MI framework to our research project

#### 2.2.1 Inclusion criteria, outcomes and further definitions.

**Overall inclusion criterion of the research project:** For our research project, encounters had to fulfil the POLAR_MI-wide inclusion criteria listed in section 2.1.1. In addition, encounters had to have at least one documented 7-character Anatomical Therapeutic Chemical (ATC) code. Encounters fulfilling these criteria constituted our research project population.

**Outcomes and outcome-related inclusion criteria:** We considered two clinically relevant outcomes: (1) documented upper gastrointestinal (GI) bleeding and perforations as an AE and (2) documented drug-related hypoglycaemia as an ADE. Subsequently, the outcomes are only called “GI bleeding” and “drug-related hypoglycaemia”. The classification as “clinically relevant” was based on an expert consensus process [23]. The decision in favour for these two outcomes was made as they could be defined by different CDS resources, thus enabling the evaluation of the feasibility of regression modelling in general and the impact of including laboratory values in such analyses. In more detail, GI bleeding was defined based on diagnoses according to the German Modification of the International Statistical Classification of Diseases and Related Health Problems (10th Revision; ICD-10-GM). Hypoglycaemia was defined via laboratory values using the Logical Observation Identifiers Names and Codes (LOINC). To assess the impact of considering the AE hypoglycaemia as drug-related, encounters had to have at least one documented antihyperglycaemic drug. Encounters fulfilling this additional inclusion criterion constituted the population considered for analyses related to the outcome drug-related hypoglycaemia. There was no additional inclusion criterion for the outcome GI bleeding, so that the population used for analyses related to this outcome was identical to the overall population of our research project. For convenience, we refer to each of these outcome-related populations as the “study population” for the respective outcome. The exact definitions of the outcomes and the outcome-related inclusion criteria are provided in Table A in S2 File.

**Further variable definitions:** Besides the variables covered by the inclusion criteria and the outcomes, we required additional variables related to specific medications, diseases, and laboratory values as covariates for the regression models (see section 2.2.2). The definitions and related assumptions for all variables are given in Table A in S2 File. In general, only documented information on the variables could be used.

**Additional preparations:** We reviewed all applied classification systems for code changes between 2018 and 2021. Due to the heterogeneity in the presentation of laboratory values across centres, it was additionally necessary to provide information on unit conversion, as presented in Table B in S2 File.

#### 2.2.2 Statistical EHR data analysis via meta-analysis.

**Population description:** We provide population descriptions for both study populations. Relevant encounter characteristics are summarised as median and relative frequency, respectively, with 95% confidence interval (95% CI), accompanied by the number of encounters having information on the respective characteristic. The description is provided overall as well as stratified by the respective outcome. Note, that the frequencies of outcomes and distributions of covariates were assessed both as simply documented and considering the chronology of covariate and outcome, i.e., the covariate value had to be documented on admission and the outcome only during the hospital stay (see Table A in S2 File).

**Regression modelling:** We applied uni- and multivariable logistic regression modelling for each binary outcome to assess its association with selected covariates. The covariates were selected to account for both varying strength of association with the respective outcome and several underlying CDS resources, in order to assess the feasibility of regression modelling in the context of distributed analysis. For each model, encounters were excluded if they had a missing value for at least one variable in the respective model. The meta-analysis was based on the locally derived regression coefficients and the related variance-covariance structure, so that we received the pooled results for the complete regression model. Besides the univariable regression models for each covariate, we built two multivariable models for each outcome: the “base model” and the “extended model”. The base model comprised demographics and information derived from medications and diagnoses, while the extended model additionally included laboratory values, which were expected to be more difficult to obtain across centres. The covariates defining the regression models are provided in Table 2. Note that the chronology of covariates and outcomes was not considered for regression modelling (for reasons, see results section 3.3.2) and, thus, the information on the outcome and the covariates only had to be documented. For each meta-analysed model, we provide the number of encounters included in the model, the (adjusted) odds ratios (OR) with 95% CI as well as the Akaike-Information-Criterion (AIC), the Bayesian-Information-Criterion (BIC), and the I^2^ statistic to measure heterogeneity. In addition to the number of encounters included in each model, we provide for each variable the number of encounters with missing information for the respective variable and an overview of the patterns of missing information across all variables (including the frequencies of their occurrences). Furthermore, we summarise local model performance indices for each model. Namely, we provide the proportion of centres with likelihood ratio (LR) test p-value below 0.05, the median area under the receiver operating characteristic curve (ROC AUC) and the median variance inflation factor (VIF). The median is accompanied by the first and third quartile (Q1, Q3).

**Table 2:** Covariates included in the base and the extended model, respectively, for each outcome.

Covariates in …	Outcome	
	GI bleeding	Drug-related hypoglycaemia
… base model	Inclusion of age, in 10 years gender NSAID ASA SSRI bisphosphonate liver disease	Inclusion of age, in 10 years gender any insulin long-acting insulin heart failure type of DM
… extended model	Base model extended by AST increased ALT increased serum albumin decreased haemoglobin decreased creatinine, in mmol/L	Base model extended by serum albumin decreased creatinine, in mmol/L

The exact definitions are provided in Tables A and B in S2 File. Abbreviations: ALT, alanine transaminase; ASA, acetylsalicylic acid; AST, aspartate aminotransferase; DM, diabetes mellitus; GI, gastrointestinal; NSAID, non-steroidal anti-inflammatory drug; SSRI, selective serotonin reuptake inhibitor.

**Definition of centre subgroups:** For each centre, local results were checked for plausibility. For the population description, the number of encounters as well as median and relative frequencies were compared to expectation and literature. In case of inconsistencies, results were checked and explanations searched. For regression models, results were checked for numerical stability. Stability was assumed if the standard errors (SE) were reasonable, i.e., the magnitude of the SE was not larger than the magnitude of the coefficient. Only numerically stable multivariable regression models were considered for the subsequent analyses. As numerically stable estimations of both models were not possible at each centre, several meta-analyses based on different centre subgroups were performed. The definitions of these analyses are provided in Table 3. Based on these analyses, the impact of including different number of centres can be analysed by comparing the analyses (B1.a) and (H1.a) with the analyses (B1.b) and (H1.b), respectively. The influence of including laboratory values in multivariable models can be assessed by comparing the results of the base model and the extended model within (B1.b) and (H1.b). The analyses (B1.c) and (H1.c) were constructed to examine how the results are affected when the chronology of covariate and outcome is considered. Therefore, these analyses were based on the centres that were also included in the analyses (B1.a) and (H1.a), respectively.

**Table 3:** Definitions of the analyses for both outcomes.

Analysis	Inclusion criteria	
	multivariable model(s)	chronology
*Outcome: GI bleeding*		
(B1.a)	base	none
(B1.b)	base, extended	none
(B1.c)^*1^		yes
*Outcome: Drug-related hypoglycaemia*		
(H1.a)	base	none
(H1.b)	base, extended	none
(H1.c)^*1^		yes

For each analysis, all centres with stable results for the respective models were included, if not stated otherwise. * ^**1**^ Same centres as in (B1.a) and (H1.a), respectively. Abbreviation: GI, gastrointestinal.

## 3. Results

### 3.1 Availability of local results for meta-analysis

**Fig 2:**
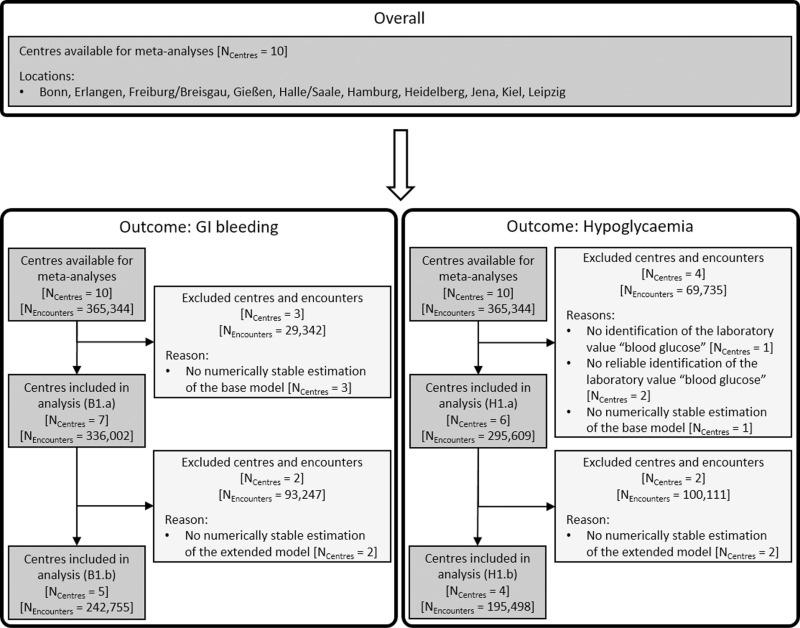
Flowchart visualising the inclusion of centres and encounters within the analyses. Numbers of included and excluded centres (N_Centres_) are provided, with reasons for exclusion. In addition, the numbers of encounters (N_Encounters_) constituting our research project population in the related analyses are provided. The definitions of the analyses are given in Table 3.

The number of centres and corresponding encounters constituting the research project populations for the defined analyses (Table 3) are given in Fig 2. Overall, for both outcomes, the requirement of laboratory values was a frequent limiting factor for receiving stable results from regression modelling. As the latter formed the basis for the inclusion of a centre into the respective meta-analysis, this circumstance led to the exclusion of centres.

### 3.2 Availability of encounters for meta-analysis

For the outcome GI bleeding, there was no outcome-related inclusion criterion, so that the study population for this outcome was identical to the population of our research project (see Fig A in S3 File). The hypoglycaemia-related inclusion criterion of at least one documented antihyperglycaemic drug, required for this AE to be considered as drug-related, induced the exclusion of about 85% of the encounters (see Fig B in S3 File). For example, in the analysis (H1.a), out of the 295,609 encounters that constituted the research project population, 44,101 encounters remained in the study population (see Fig B in S3 File). Besides these encounter exclusions, missing laboratory values were the most common reason for encounter exclusions for both outcomes (see Figs A and B in S3 File). The proportion of encounters with missing laboratory values can be directly assessed by moving from the study population to the population underlying the extended model estimation for the outcome GI bleeding in the analysis (B1.b). There, the number of encounters was reduced by approximately 75% due to missing laboratory values (see Fig A in S3 File). For the outcome drug-related hypoglycaemia, the extent of the impact of a missing blood glucose laboratory value cannot be exactly quantified, as it was one of several reasons for excluding encounters when moving from the study population to the base model population (see Fig B in S3 File). However, requiring laboratory values other than blood glucose in the analysis (H1.b) almost halved the size of the extended model population compared to the size of the base model population (see Fig B in S3 File). Details on the pattern of missing information, i.e., the interplay of different variables (outcomes, independent variables included in the regression models) in inducing a specific constellation of missing information, are provided in Tables C–H in S2 File.

### 3.3 Frequencies of outcomes and distributions of covariates

In terms of outcome frequencies and covariate distributions, feasibility included two aspects: (1) availability of the respective information and (2) plausible distributions across encounters with the available information. Here we provide the meta-analysed population descriptions, including the impact of considering laboratory values and the chronology of events. The related plausibility of the distributions is examined in the discussion section.

**Fig 3:**
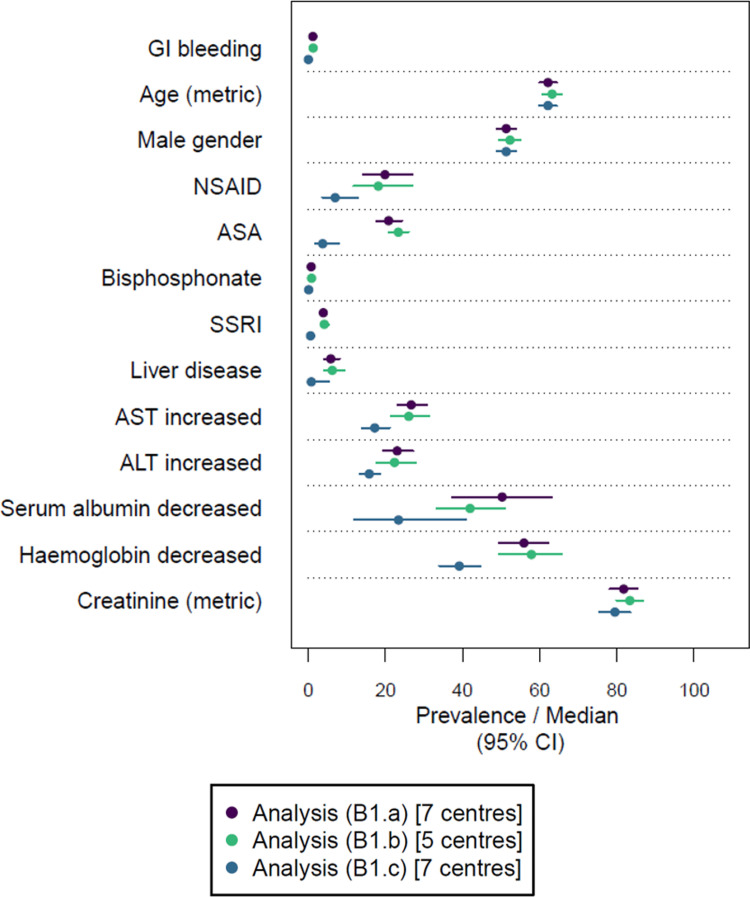
Meta-analysed description of encounter characteristics for the outcome GI bleeding in the study population. Prevalence is provided for categorical variables and median values for metric variables (only age in years and creatinine in µmol/L). Estimates are accompanied by 95% confidence intervals (CI). Descriptions are provided for the analyses (B1.a), (B1.b) and (B1.c). Note that the same centres were included in the analyses (B1.a) and (B1.c). The complete results of the cohort descriptions are provided in Tables I–K in S2 File. The definitions of the analyses are provided in Table 3. Further abbreviations: ALT, alanine transaminase; ASA, acetylsalicylic acid; AST, aspartate aminotransferase; GI, gastrointestinal; NSAID, non-steroidal anti-inflammatory drug; SSRI, selective serotonin reuptake inhibitor.

In the analysis (B1.a), we found that 1.2% (95% CI: 0.8% to 1.7%) of encounters had a documented GI bleeding (see Fig 3). A liver disease was documented for 5.8% (4.2%, 8.1%) of all encounters. In terms of medications, the frequency of a documented NSAID was lower in the subgroup of encounters with a documented GI bleeding compared to encounters without a documented GI bleeding. Among laboratory values, decreased haemoglobin was the most common abnormal laboratory value, documented in 55.9% (49.4%, 62.2%) of all encounters. The complete meta-analysed cohort description for the analysis (B1.a) is provided in Table I in S2 File.

**Fig 4:**
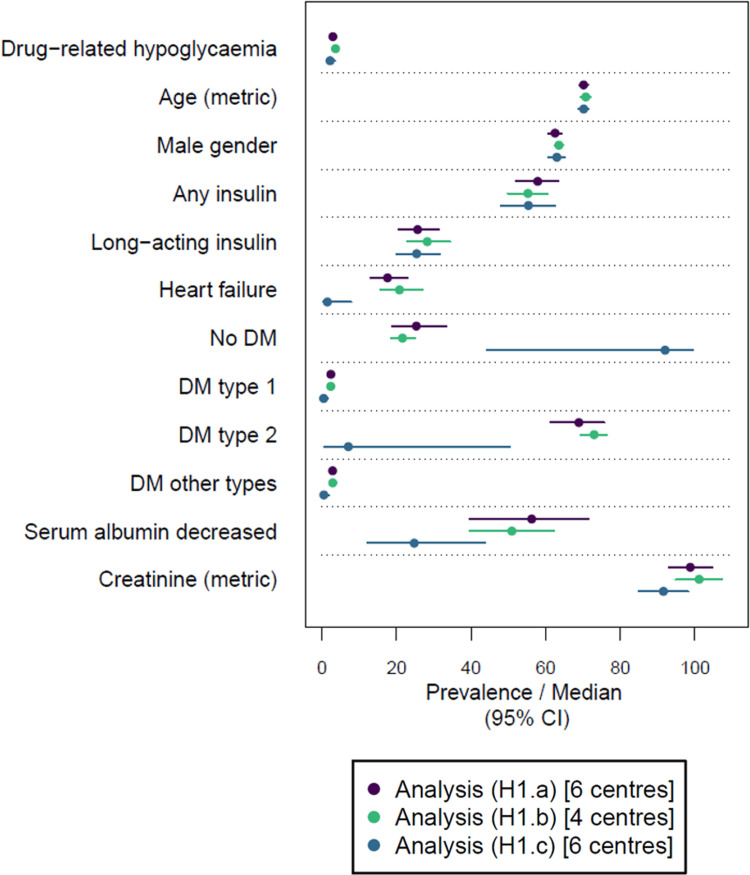
Meta-analysed description of encounter characteristics for the outcome drug-related hypoglycaemia in the study population. Prevalence is provided for categorical variables and median values for metric variables (only age in years and creatinine in µmol/L). Estimates are accompanied by 95% confidence intervals (CI). Descriptions are provided for the analyses (H1.a), (H1.b) and (H1.c). Note that the same centres were included in the analyses (H1.a) and (H1.c). The complete results of the cohort descriptions are provided in Tables L–N in S2 File. The definitions of the analyses are provided in Table 3. Further abbreviation: DM, diabetes mellitus.

In the analysis (H1.a), 2.9% (2.2%, 4.0%) of encounters exhibited the outcome drug-related hypoglycaemia (see Fig 4). Although the inclusion criterion was the documentation of at least one antihyperglycaemic drug, 25.3% (18.8%, 33.3%) of encounters had no documented diabetes mellitus (DM). Type 1, type 2, and other types of DM were documented for 2.4% (1.9%, 3.0%), 68.9% (61.2%, 75.7%) and 2.9% (2.3%, 3.6%) of encounters, respectively. The complete meta-analysed cohort description of the analysis (H1.a) is provided in Table L in S2 File.

#### 3.3.1 Influence of considering laboratory values.

The point estimates of the prevalences observed in the analyses (B1.a) and (H1.a) were similar to those found in the analyses (B1.b) and (H1.b), respectively, in which less centres were included due to the consideration of laboratory values (see Figs 3 and 4). The complete meta-analysed cohort descriptions for the analyses (B1.b) and (H1.b) are provided in Tables J and M in S2 File, respectively.

#### 3.3.2 Influence of considering the chronology of events.

The comparisons between the analyses (B1.a) and (B1.c) as well as the analyses (H1.a) and (H1.c) allow the examination of consequences when considering the chronology of events, i.e., the covariates’ timestamp had to be identical to the day of admission (day 1 of hospital stay) and the outcomes’ timestamp had to be between day 2 of hospital stay and the day of discharge.

When considering the chronology of events, the prevalence estimates of both outcomes and all covariates decreased (see Figs 3 and 4). For example, a GI bleeding was documented during hospital stay for only 0.02% (0.01%, 0.06%) of encounters according to the analysis (B1.c), corresponding to a reduction of encounters with a documented GI bleeding of approximately 98% compared to the analysis (B1.a), highlighting that most GI bleedings were present on admission, rather than hospital-onset GI bleedings (see Tables I and K in S2 File). For the outcome drug-related hypoglycaemia, the inclusion criterion was less frequently fulfilled. As a result, less than 50% of the encounters that could be included in the analysis (H1.a) could be included in the analysis (H1.c) (see Tables L and N in S2 File). In addition, the proportion of encounters with missing blood glucose information increased in the analysis (H1.c). Due to the substantial sample size reduction observed for both outcomes when considering the chronology of events, regression modelling considering this chronology was not yet performed.

### 3.4 Regression analyses

**Fig 5:**
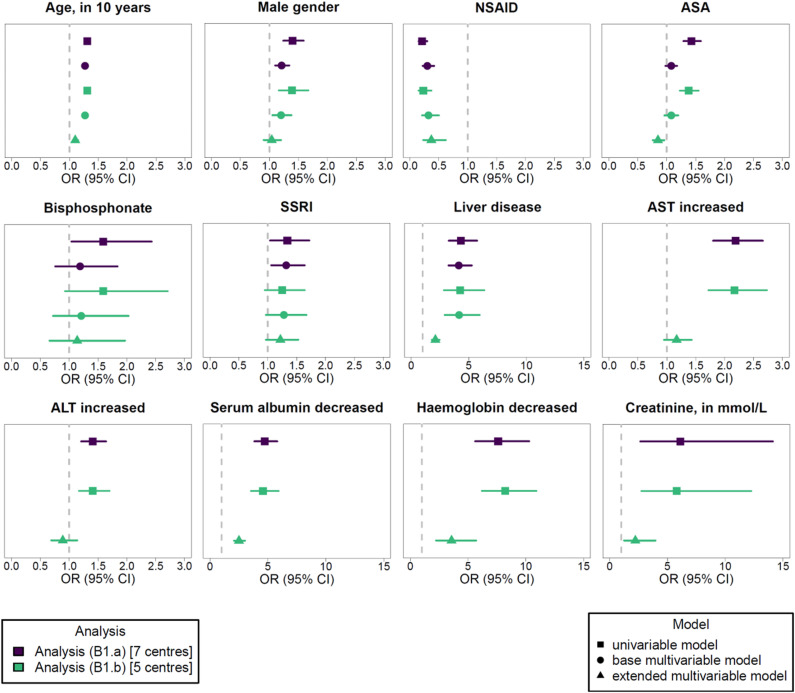
Meta-analysed regression modelling results for the outcome GI bleeding. Odds ratios (OR) with 95% confidence intervals (CI) are given. Results are provided for the analyses (B1.a) and (B1.b). The results of the univariable and multivariable models are provided stratified by the variables included in the extended model. The OR of 1 is indicated by the dashed line. Note that laboratory values were only included in the extended model. The complete results of the regression modelling are provided in Tables O and P in S2 File. The definitions of the analyses are provided in Table 3. Further abbreviations: ALT, alanine transaminase; ASA, acetylsalicylic acid; AST, aspartate aminotransferase; NSAID, non-steroidal anti-inflammatory drug; SSRI, selective serotonin reuptake inhibitor.

**Fig 6:**
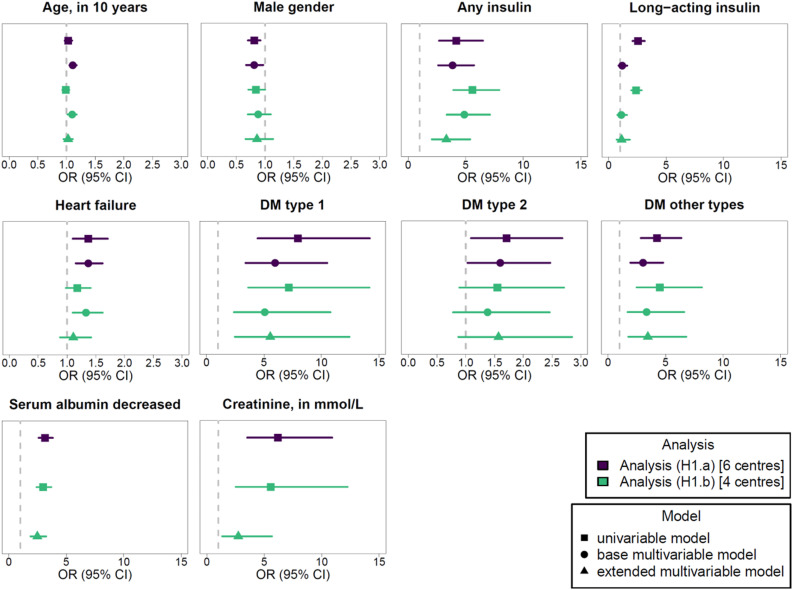
Meta-analysed regression modelling results for the outcome drug-related hypoglycaemia. Odds ratios (OR) with 95% confidence intervals (CI) are given. Results are provided for the analyses (H1.a) and (H1.b). The results of the univariable and multivariable models are provided stratified by the variables included in the extended model. The OR of 1 is indicated by the dashed line. Note that laboratory values were only included in the extended model. The complete results of the regression modelling are provided in Tables Q and R in S2 File. The definitions of the analyses are provided in Table 3. Further abbreviation: DM, diabetes mellitus.

In our project, the feasibility of regression modelling included two aspects: (1) stable regression model estimation and (2) regression results consistent with the literature. We will come back to the latter aspect in the discussion section. Here, we present the meta-analysed regression results and the observed impact of including laboratory values. The results of the meta-analysed uni- and multivariable regression analyses for the outcomes GI bleeding and drug-related hypoglycaemia are given and illustrated in Figs 5 and 6, respectively.

In the analyses (B1.a) and (H1.a), all selected diagnoses, laboratory values and medications, except of NSAID, were univariably associated with a higher chance of exhibiting the respective outcome. In the multivariable base model of analysis (B1.a), SSRI and liver disease remained associated with a higher chance of exhibiting a GI bleeding, while for the outcome drug-related hypoglycaemia, any insulin, heart failure and all types of DM remained associated with this outcome in the analysis (H1.a). Encounters with a documented NSAID were less likely to exhibit a documented GI bleeding in all univariable and multivariable models, a seemingly implausible result which will be discussed in section 4.1. The complete meta-analysed regression modelling results of the analyses (B1.a) and (H1.a), including detailed information on the observed substantial heterogeneity (I^2^ > 75%), are provided in Tables O and Q in S2 File.

#### 3.4.1 Influence of considering laboratory values.

When adjusting for laboratory values in the extended models of the analyses (B1.b) and (H1.b) and compared with the base models of the same analyses (see Figs 5 and 6), evidence for the associations of male gender with GI bleeding as well as of age and heart failure with drug-related hypoglycaemia were no longer observed in the extended models. In contrast, after adjusting for laboratory values, encounters with a documented ASA were less likely to exhibit a documented GI bleeding. Regarding the laboratory values themselves, all included abnormal laboratory values were univariably associated with a larger chance of having the related outcome documented. In the extended model of the outcome GI bleeding, we no longer observed evidence for an association with increased AST and ALT. For both outcomes, the heterogeneity was moderate for the extended models. The complete meta-analysed regression modelling results of the analyses (B1.b) and (H1.b) are provided in Tables P and R in S2 File.

#### 3.4.2 (Local) model performance.

We also assessed selected model performance measures (see Tables O–R in S2 File). To answer the question of whether the model including covariates had locally a better fit than the intercept-only model, the likelihood-ratio (LR) test was used. A better fit for the model with covariates was indicated, if the related p-value was below 0.05. For some univariable models, the p-value of the local LR test was not below 0.05 in all centres, even though the covariate was associated globally (in the related meta-analysis) with the respective outcome. For example, when considering the variable bisphosphonates in the analysis (B1.a), this variable was associated with the outcome GI bleeding in the meta-analysis, but the p-value of the local LR tests was only in one centre below 0.05. For the multivariable models, the local LR test results coincided across all centres (all LR test p-values below 0.05). When comparing the multivariable base model and the multivariable extended model within one analysis, AIC and BIC calculated for the meta-analysed regression models indicated a better fit for the base model. Comparing the first quartile of the locally assessed ROC AUC of the extended model to the base model for both outcomes, the extended model exhibited larger values. In general, for the outcome GI bleeding, the extended model of the analysis (B1.b) exhibited a first quartile of the ROC AUC above 0.7. For the outcome drug-related hypoglycaemia, this held for all multivariable models.

## 4. Discussion

Within our research project, we aimed to investigate the feasibility of distributed regression modelling for detecting and predicting upper GI bleeding and drug-related hypoglycaemia using routine healthcare data from German university hospitals. We applied the POLAR_MI ETL Pipeline at the DIC of the respective hospitals, evaluating patient characteristics and estimating numerically stable regression models in a privacy-compliant manner via the MII core data set (CDS) without direct data access for the analysts. To achieve this, we tailored our research question to the available data by questioning the integration of the chronology of events and by using a step-by-step approach for regression modelling. For the latter, we started with univariable models, moved to a model including demographics, medications and diagnoses, and finally to the desired model adding laboratory values.

### 4.1 Plausibility of results without considering laboratory values or the chronology of events

The demographics of the study population for the outcome GI bleeding can be compared to the German hospital statistics for the year 2022, showing that the distributions of age and gender were comparable [24]. The prevalences of the documented diagnoses GI bleeding and liver disease were also comparable to those in the literature [25,26]. For the outcome drug-related hypoglycaemia, applying the outcome-specific inclusion criterion of taking at least one antihyperglycaemic drug, which can be seen as a proxy for having DM, resulted in an older study population comprising more men than women. This is consistent with the literature on the demographics of patients with DM in Germany [27].

As all selected covariates are discussed in the literature as risk factors for the respective outcome, the fact that all selected diagnoses, laboratory values and medications, with the exception of NSAID, were at least univariably associated with a higher chance of the respective outcome demonstrates the feasibility of regression modelling (in terms of effect direction, effect size and statistical power) [28–36]. For the outcome GI bleeding, decreased haemoglobin had the strongest association of all covariates, which is in line with expectations and the current literature, as decreased haemoglobin is the physiological consequence of GI bleeding [37,38]. For the outcome drug-related hypoglycaemia, type 1 DM had the strongest association, which is also in line with the current medical knowledge, as hypoglycaemia is more common in patients with type 1 DM than in those with type 2 DM [36,39,40]. Covariates that are more controversial in the literature regarding their association with the outcome, e.g., SSRI and bisphosphonates for GI bleeding or female gender for drug-related hypoglycaemia, only showed an association in analyses with more centres, i.e., analyses with a larger sample size and thus more statistical power [30,32,34,41].

Among the medications evaluated, encounters with a documented NSAID were less likely to exhibit a documented GI bleeding in all analyses (estimated prevalences and regression modelling), being the only seemingly unplausible result [42–44]. A possible explanation for this observation is that most of the observed GI bleedings may have been present on admission. Therefore, NSAID may not have been administered during the hospital stay or may have been discontinued on admission prior to any medication documentation in patients admitted with GI bleeding, as drug labels list previous or acute GI bleedings as contraindications of NSAID, recommending their discontinuation [45,46].

### 4.2 Consideration of laboratory values

When integrating laboratory values into an analysis, there are several aspects to consider regarding their missing values. First, there are different prerequisites for requesting a specific laboratory value (e.g., ward type, medical specialty, comorbidity), meaning that not every laboratory value is requested for every encounter. Second, the definition of “missing information” differs from that used for diagnoses, for example. For diagnoses, information for a specific disease is missing if the respective FHIR resource is unavailable or empty. For laboratory values, such a general assessment of missing information is not possible. The decision must be made separately for each laboratory value. The individual decision is based on the absence of any of the specified LOINC codes for that specific laboratory value in the CDS data. For example, if no blood glucose value was obtained for an encounter, we categorised the encounter as having missing information on blood glucose. Finally, missing values could be influenced by laboratory systems not connected to the DIC, difficulties in mapping internal coding to LOINC codes, and unexpected LOINC codes or units, despite our attempts in unit conversion and overarching LOINC code specification. These circumstances led to varying amounts of encounters with missing values among the laboratory values considered. However, when comparing the study populations, the exclusion of centres due to missing laboratory values or unstable extended model estimation (including laboratory values) seems to have had no substantial effect on the distribution of outcomes and covariates, which may indicate that the centres were not that different in terms of their cohort characteristics. Overall, the prevalence of decreased haemoglobin stood out, with over 50% of encounters being anaemic, which is in line with the literature as we used the World Health Organisation’s high threshold [47].

Despite the missing values, regression modelling with laboratory values was possible and resulted in plausible results. The effect directions in the base models did not differ between analyses requiring laboratory values and those without (comparisons: base model between the analyses (B1.a) and (B1.b) as well as between (H1.a) and (H1.b)). After the adjustment for laboratory values in the extended models, only the associations of male gender with GI bleeding as well as of age and heart failure with drug-related hypoglycaemia were no longer observed, probably because of reduced statistical power due to the lower number of encounters included. The enzymes AST and ALT, which indicate liver diseases, exhibited an association with GI bleeding in the univariable models, but not in the multivariable model when adjusted for liver disease, which is reasonable [31]. To summarise, the strengths and directions of the associations were mostly maintained when adjusting for laboratory values that caused a reduction in sample size.

### 4.3 Consideration of the chronology of events

We have made several attempts to incorporate the chronology of covariates and outcomes in the regression models. This kind of analysis requires precise, interpretable timestamps. In our case, the requirement for such timestamps led to a substantial reduction in sample size, so we did not pursue regression modelling considering timestamps in this work, but we plan to return to this issue in the ongoing follow-up project called INTERPOLAR (INTERventional POLypharmacy-drug interActions-Risks) [48].

Possible reasons for our observations are manifold. First, reimbursement of treatments in Germany is based on coded diagnoses. The data for this purpose are coded retrospectively, mostly after discharge, without specifying whether the coded condition was present on admission or occurred during the hospital stay [49,50]. Therefore, although diagnoses may have been present on admission, their timestamps are often delayed. For example, liver disease, heart failure and DM are chronic conditions and their reduction in prevalence when assessed only on day 1 of hospitalisation cannot be explained by diagnoses first identified during the stay. Second, ambiguous filling of mandatory timestamps occurred. For example, in the case of laboratory values, identical timestamps were provided for sampling, ordering the service, sample receipt, analysis and result transmission. Third, the same event was coded more than once at different times, e.g., a GI bleeding was coded on admission and during hospital stay. Finally, timestamps were sometimes set to unknown or were not present.

### 4.4 Implications for predictive modelling

We estimated multivariable regression models with a median ROC AUC above 0.7. Thus, our original purpose of building models to predict ADE seems possible under certain conditions. However, the decision on which outcomes and independent variables to include in the regression models must always be made weighing medical plausibility, predictive value and sufficient statistical power, as the unavailability of certain variables can induce a considerable loss in sample size and statistical power. Interestingly, when summarising the local ROC AUC values, a better predictive performance was indicated for the extended models considering laboratory values, but the AIC and BIC for the meta-analysed regression models were better for the models without laboratory values, indicating that the penalty term for more variables dominated.

When developing models to predict ADE, a development and a validation dataset are required. Based on the data within the MII, there are generally two approaches to obtain these two datasets. First, a subset of centres (one or more) can be used for model development and the remaining centres for model validation. Second, both model development and model validation can be done in all centres if the dataset is split at a certain point in time. For example, data available at the start of the study can be used for model development and data collected during (and after) model development can be used for model validation. Of course, it is also possible to rely entirely on retrospective data, i.e., data available at study begin. Both approaches (“splitting by centre” and “splitting by time”) have advantages and disadvantages. Splitting by centre – especially if only one centre is used for model development and all others for validation – would facilitate implementation in the context of distributed analysis. Splitting by time would probably lead to more generalisable results, as the development would take place in all centres, taking into account the heterogeneity of the centres.

### 4.5 Strengths and limitations

For the first time in Germany, the feasibility of evaluating patient characteristics and regression modelling in a privacy-preserving, multicentre, distributed analysis based solely on the data interoperability standards of the MII at the DIC was investigated. We obtained plausible results from a broad dataset reflecting routine healthcare in German university hospitals, which clearly demonstrates the potential of analyses within the MII. Here, one must always bear in mind that we only evaluated two different outcomes. However, these selected outcomes reflected different CDS resources (diagnoses, laboratory values), enabling the assessment of the impact of including laboratory values. In addition, one must keep in mind that we worked with EHR data from 2018 to the end of 2021 and the related MII CDS specifications (version 1.0). In the meantime, the implementation of the CDS version 2.0 is spreading. The extent to which this will improve the potential of such analyses is being investigated in the INTERPOLAR project [48]. Furthermore, the EHR data used were coded for purposes other than research, (documentation) routines varied between centres and wards, and the available data also depended on the way of implementing the MII CDS specifications. This introduced a non-negligible amount of not randomly missing information and heterogeneity (encountered through random-effects meta-analyses) in the received local results, which was related to the reported numbers of patients, encounters, medications, diagnoses, laboratory values and missing values. In particular, missing information on laboratory values affected the statistical power through limiting the number of encounters and centres included in some meta-analyses, and the inability to integrate timestamps in the models affected their predictive value when aiming at predicting ADE based on risk factors present prior to the event. The latter impact on model fit cannot be reflected by any comprehensive evaluation of performance metrics, as missing timestamps affect the contextual value of the models in terms of chronological order. Being aware of these issues and the original purpose of the documented data, we focused in particular on (1) risk factors reflecting both the varying strength of association with the respective outcome and the availability of information based on different CDS resources, and (2) the construction of regression models of varying complexity. This allowed a reliable interpretation of the results despite the issues mentioned above, as in this way results that were not consistent with the literature and issues related to available timestamps could be explained by the data structure and data availability. This led to important insights that will also guide improvements of the MII CDS specifications, thus enhancing interoperability in Germany.

## 5. Conclusions

German inpatient treatment data from university hospitals with different HIS infrastructures were made accessible in a privacy-preserving manner for AE and ADE evaluation and regression modelling. Using a distributed analysis approach without direct data access for the analysts, we received plausible estimates for prevalence and regression modelling odds ratios. We conclude that the development of predictive models for ADE in a distributed setting is possible across many institutions if the research questions can be tailored meaningful to the infrastructure and data available.

## Supporting information

S1 File Membership list of POLAR_MI. (PDF)

S2 File Supplemental tables. (PDF)

S3 File Supplemental figures. (PDF)
