# Supplementary material for: Challenges in detecting and predicting adverse drug events via distributed analysis of electronic health record data from German university hospitals
Source: PLOS Digit Health. 2025 Jun 26;4(6):e0000892. doi: 10.1371/journal.pdig.0000892 (PMC12200832; doi:10.1371/journal.pdig.0000892)
Supplement: S1 File — (PDF) [file pdig.0000892.s001.pdf]

Supporting information file S1: Membership list of POLAR_MI

Challenges in detecting and predicting adverse drug events via distributed analysis of electronic health record data from German university hospitals

Anna Maria Wermund[1], Torsten Thalheim[2,3,4], André Medek[5], Florian Schmidt[3], Thomas Peschel[3], Alexander Strübing[3], Daniel Neumann[3], André Scherag[6], Markus Loeffler[3], Miriam Kesselmeier[6,¶] and Ulrich Jaehde[1,¶*] for the POLAR_MI Consortium[+]

[+] The membership list of POLAR_MI is provided in Supporting information file S1 (“Membership list of POLAR_MI”)

[1] Department of Clinical Pharmacy, Institute of Pharmacy, University of Bonn, Bonn, Germany.
[2] Interdisciplinary Centre for Bioinformatics, Leipzig University, Leipzig, Germany
[3] Institute for Medical Informatics, Statistics and Epidemiology (IMISE), Leipzig University, Leipzig, Germany
[4] Deutsches Biomasseforschungszentrum gGmbH, Torgauer Str. 116, 04347 Leipzig, Germany
[5] Medical & Scientific Technology Development & Coordination (MWTek), University Hospital Bonn, 53127 Bonn, Germany
[6] Institute of Medical Statistics, Computer and Data Sciences (IMSID), Jena University Hospital – Friedrich Schiller University Jena, Jena, Germany

¶ Equal contribution

* u.jaehde@uni-bonn.de

Table S. Membership list of POLAR_MI. Members on this list explicitly opt-in. Each member on this list provided the respective information themselves.

First name	Last name	ORCID	Affiliation (English)	Affiliation (German)
Lars-Christian	Achauer		Medical Data Integration Center, University Hospital Tübingen, Tübingen, Germany	Universitätsklinikum Tübingen
Fady	Albashiti			Ludwig-Maximilians-Universität München
Danny	Ammon	0000-0001-8960-7316	Data Integration Center; Jena University Hospital; Jena, Germany	Datenintegrationszentrum; Universitätsklinikum Jena; Jena
Wahram	Andrikyan	0000-0002-4885-9864	Institute of Experimental and Clinical Pharmacology and Toxicology, Friedrich-Alexander-Universität Erlangen-Nürnberg, Erlangen, Germany	Institut für Experimentelle und Klinische Pharmakologie und Toxikologie, Friedrich-Alexander-Universität Erlangen-Nürnberg, Erlangen
Jördis	Beck	0009-0007-3826-9047	Data Integration Center, Institute of Medical Informatics, Justus Liebig University, Giessen, Germany	Datenintegrationszentrum, Institut für Medizinische Informatik, Justus Liebig Universität, Gießen, Deutschland
Björn	Bergh			Christian-Albrechts-Universität zu Kiel
Oya	Beyan			RWTH Aachen
Stephanie	Biergans	0000-0002-0120-1301	Medical Data Integration Center, University Hospital Tübingen, Tübingen, Germany	Universitätsklinikum Tübingen
Harald	Binder			Universitätsklinikum Freiburg
Romina	Blasini		University Hospital Giessen and Marburg, location Giessen, Germany	Universitätsklinikum Gießen und Marburg, Standort Gießen, Deutschland
Martin	Boeker	0000-0003-2972-2042	Chair of Medical Informatics, Institute of Artificial Intelligence and Informatics in Medicine, TUM University Hospital, Munich, Germany	TU München
Ruwen	Böhm	0000-0003-1007-3011	Institute of Experimental and Clinical Pharmacology, University Hospital Schleswig-Holstein, Germany	Institut für Experimentelle und Klinische Pharmakologie, Universitätsklinikum Schleswig-Holstein, Campus Kiel

Julian	Brandes			Universitätsklinikum Leipzig
Claudia	Bulin	0009-0000-6008-4574	Institute of Experimental and Clinical Pharmacology, University Hospital Schleswig-Holstein, Campus Kiel, Germany	Institut für Experimentelle und Klinische Pharmakologie, Universitätsklinikum Schleswig-Holstein, Campus Kiel
Ingolf	Cascorbi	0000-0002-2182-9534	Institute of Experimental and Clinical Pharmacology, University Hospital Schleswig-Holstein, Campus Kiel, Germany	Institut für Experimentelle und Klinische Pharmakologie, Universitätsklinikum Schleswig-Holstein, Campus Kiel
Martin	Coenen	0000-0003-0439-440	Institute of Clinical Chemistry and Clinical Pharmacology, University Hospital Bonn, Germany	Universitätsklinikum Bonn
Tobias	Dreischulte	0000-0003-2345-5377	Institute of General Practice and Family Medicine, LMU University Hospital, LMU Munich, Munich, Germany	Institut für Allgemeinmedizin, Klinikum der Ludwig-Maximilians-Universität München, München, Deutschland
Pauline	Dürr	0000-0001-5772-4956	Institute of Experimental and Clinical Pharmacology and Toxicology, Friedrich-Alexander-Universität Erlangen-Nürnberg, Erlangen, Germany<br><br>Pharmacy Department, Erlangen University Hospital, Erlangen, Germany	Institut für Experimentelle und Klinische Pharmakologie und Toxikologie, Friedrich-Alexander-Universität Erlangen-Nürnberg, Erlangen<br><br>Apotheke des Universitätsklinikums Erlangen, Universitätsklinikum Erlangen, Erlangen, Deutschland
Axel	Dürrbeck			Apotheke des Universitätsklinikum Halle, Universitätsklinikum Halle, Halle, Deutschland
Andreas	Dürschmid			Universität Leipzig
Albrecht	Eisert			Universitätsklinikum Aachen
Felix	Erdfelder			Universitätsklinikum Bonn

Katrin	Farker		Hospital Pharmacy, University Center for Pharmacotherapy and Pharmacoeconomics, Jena University Hospital, Jena, Germany	Apotheke des Universitätsklinikums Jena, Universitäres Zentrum für Pharmakotherapie und Pharmakoökonomie (UZP), Universitätsklinikum Jena, Jena, Deutschland
Martin	Federbusch	0000-0002-1126-5763	1.) Institute of Laboratory Medicine, Clinical Chemistry and Molecular Diagnostics, University of Leipzig Medical Center, Leipzig, Germany<br>2.) Medical Informatics Center - Dept. for Clinical AI and Translational Medicine, University of Leipzig Medical Center	1.) Institut für Laboratoriumsmedizin, Klinische Chemie und Molekulare Diagnostik, Universitätsklinikum Leipzig, Leipzig, Deutschland<br>2.) Medizininformatikzentrum - Abteilung für medizinische KI und Translation, Universitätsklinikum Leipzig
Steffen	Franke			Universitätsklinikum Leipzig
Norman	Freier			Universitätsklinikum Hamburg-Eppendorf
Thomas	Frese			Universitätsklinikum Halle
Fleur	Fritz-Kebede	0000-0002-9005-6766	Institute of Medical Informatics, Heidelberg University Hospital	Institut für Medizinische Informatik, Universitätsklinikum Heidelberg
Martin F.	Fromm	0000-0002-0334-7478	Institute of Experimental and Clinical Pharmacology and Toxicology, Friedrich-Alexander-Universität Erlangen-Nürnberg, Erlangen, Germany	Institut für Experimentelle und Klinische Pharmakologie und Toxikologie, Friedrich-Alexander-Universität Erlangen-Nürnberg, Erlangen
Thomas	Ganslandt	0000-0001-6864-8936	Friedrich-Alexander-Universität Erlangen-Nürnberg, Medical Informatics, Erlangen, Germany	Friedrich-Alexander-Universität Erlangen-Nürnberg, Medizinische Informatik, Erlangen, Deutschland
Jan Erik	Gewehr	0009-0006-6453-9082	Business Unit for Information Technology, University Medical Centre Hamburg Eppendorf, Martinistraße 52, 20246 Hamburg	Universitätsklinikum Hamburg-Eppendorf
Daniel	Grigutsch			Universitätsklinikum Bonn
Udo	Hahn	0000-0002-5052-0245	Jena University Language &	Friedrich-Schiller-Universität Jena

			Information Engineering Lab (JULIE Lab), Friedrich-Schiller-Universität Jena, Fürstengraben 30, D-07743, Jena	
Annette	Härdtlein	0009-0006-5611-1270	Institute of General Practice and Family Medicine, LMU University Hospital, LMU Munich, Munich, Germany	Institut für Allgemeinmedizin, Klinikum der Ludwig-Maximilians-Universität München, München, Deutschland
Ralf	Harnisch			Apotheke des Universitätsklinikum Halle, Universitätsklinikum Halle, Halle, Deutschland
Steffen	Härterich	0000-0003-2395-1522	Hospital Pharmacy, University Medical Centre Hamburg Eppendorf, Martinistrasse 52, 20246 Hamburg, Germany	Universitätsklinikum Hamburg-Eppendorf
Renate	Häuslschmid			Universitätsklinikum Freiburg
Christian	Haverkamp	0000-0001-8165-4783	Institute of Digitalization in Medicine, Faculty of Medicine and Medical Center, University of Freiburg, Freiburg	Uniklinik Freiburg
Oliver	Heinze			Universitätsklinikum Heidelberg
Petar	Horki			Uniklinik Freiburg
Martin	Hug			Universitätsklinikum Freiburg
Tanja	Iskra			Universitätsklinikum Bonn
Ulrich	Jaehde	0000-0002-2493-7370	Department of Clinical Pharmacy, Institute of Pharmacy, University of Bonn, 53121 Bonn, Germany	Pharmazeutisches Institut, Abteilung Klinische Pharmazie, Universität Bonn
Simon	Jäger		Department of Clinical Pharmacology, University Hospital Tuebingen, 72076 Tübingen, Germany<br>Institute of Clinical Pharmacology, Klinikum Nürnberg, 90419 Nürnberg, Germany	Abteilung Klinische Pharmakologie Universitätsklinikum Tübingen, 72076 Tübingen<br>Institut für Klinische Pharmakologie, Klinikum Nürnberg, 90419 Nürnberg
Patrick	Jürs			Universitätsklinikum Hamburg-Eppendorf

Christian Philipp	Jüttner		Department of Clinical Pharmacology, University Hospital Tuebingen, 72076 Tübingen, Germany	Abteilung Klinische Pharmakologie Universitätsklinikum Tübingen, 72076 Tübingen
Jenny	Kaftan			Universität Leipzig LIFE MC
Thorsten	Kaiser			Universitätsklinikum Leipzig
Katharina	Karsten Dafonte	0000-0002-2239-8860	Institute of Clinical Chemistry and Clinical Pharmacology, University Hospital Bonn, Germany	Universitätsklinikum Bonn
Miriam	Kesselmeier	0000-0001-6462-2579	Institute of Medical Statistics, Computer and Data Sciences (IMSID); Jena University Hospital; Jena; Germany	Institut für Medizinische Statistik, Informatik und Datenwissenschaften (IMSID); Universitätsklinikum Jena; Jena; Deutschland
Saskia	Kiefer			Universitätsklinikum Freiburg
Sophia	Klasing			Universitätsklinikum Heidelberg
Oliver	Kohlbacher	0000-0003-1739-4598		Universitätsklinikum Tübingen
Helene	Köster		Erlangen University Hospital, Medical Center for Information and Communication Technology, Erlangen, Germany	Universitätsklinikum Erlangen, Medizinisches Zentrum für Informations- und Kommunikationstechnologie, Erlangen, Deutschland
Detlef	Kraska	0000-0003-2174-2532	Erlangen University Hospital, Medical Center for Information and Communication Technology, Erlangen, Germany	Universitätsklinikum Erlangen, Medizinisches Zentrum für Informations- und Kommunikationstechnologie, Erlangen, Deutschland
Sascha	Krause			Universitätsklinikum Leipzig
Sarah	Kreutzke		Universital Hospital Aachen, location Aachen, Germany	Universitätsklinikum Aachen
Klaus	Kuhn			Technische Universität München
Simone	Lederer			Universitätsklinikum Tübingen
Moritz	Lehne			BIH

Matthias	Löbe	0000-0002-2344-0426	Institute of Medical informatics, Statistics and Epidemiology, University Leipzig, Leipzig, Germany	Institut für Medizinische Informatik, Statistik und Epidemiologie, Universität Leipzig, Leipzig, Deutschland
Markus	Loeffler	0000-0002-0424-9933	Institute of Medical informatics, Statistics and Epidemiology, University Leipzig, Leipzig, Germany	
Christina	Lohr			Friedrich-Schiller-Universität Jena
Volker	Lowitsch			Universitätsklinikum Aachen
Matthias	Lüönd			Universitätsklinikum Heidelberg
Irina	Lutz			Universitätsklinikum Aachen
Renke	Maas	0000-0002-5498-9761	Institute of Experimental and Clinical Pharmacology and Toxicology, Friedrich-Alexander-Universität Erlangen-Nürnberg, Erlangen, Germany	Institut für Experimentelle und Klinische Pharmakologie und Toxikologie, Friedrich-Alexander-Universität Erlangen-Nürnberg, Erlangen
Jonathan	Mang	0000-0003-0518-4710	Erlangen University Hospital, Medical Center for Information and Communication Technology, Erlangen, Germany	Universitätsklinikum Erlangen, Medizinisches Zentrum für Informations- und Kommunikationstechnologie, Erlangen, Deutschland
Kurt	Marquardt			Universitätsklinikum Gießen
André	Medek	0000-0003-0569-2395	Executive Department of Medical Scientific Technology Development and Coordination (MWTek), University Hospital Bonn, 53127 Bonn, Germany	Stabsstelle Medizinisch-Wissenschaftliche Technologieentwicklung und -koordination (MWTek), Uniklinikum Bonn
Frank	Meineke	0000-0002-9256-7543	Institute of Medical informatics, Statistics and Epidemiology, University Leipzig, Leipzig, Germany	Universität Leipzig
Angela	Merzweiler			Universitätsklinikum Heidelberg
Achim	Michel-Backofen			Universität Gießen
Achim	Michel-Backofen		University Hospital Giessen and Marburg, location Giessen, Germany	Universitätsklinikum Gießen und Marburg, Standort Gießen, Deutschland

Rajesh	Murali	0009-0008-7177-3568	Department of Biomedical Informatics, University Medical Center Mannheim, Heidelberg University, Mannheim, Germany	Abteilung für Biomedizinische Informatik, Universitätsmedizin Mannheim, Universität Heidelberg, Mannheim, Deutschland
Beate	Mussawy	0000-0003-2673-3336	Hospital Pharmacy, University Medical Center Hamburg-Eppendorf, Hamburg, Germany	Universitätsklinikum Hamburg-Eppendorf
Daniel	Neumann	0000-0002-4639-5189	Institute of Medical informatics, Statistics and Epidemiology, University Leipzig, Leipzig, Germany	
Joachim	Neumann			Universitätsklinikum Halle
Christian	Niklas			Universitätsklinikum Heidelberg
Matthias	Nüchter			Universität Leipzig LIFE MC
Katharina	Oswald			Universitätsklinikum Freiburg
Julia	Palm		Institute of Medical Statistics, Computer and Data Sciences (IMSID); Jena University Hospital; Jena; Germany	Institut für Medizinische Statistik, Informatik und Datenwissenschaften (IMSID); Universitätsklinikum Jena; Jena; Deutschland
Thomas	Peschel			Universität Leipzig
Hans-Ulrich	Prokosch	0000-0001-6200-753X	Friedrich-Alexander-Universität Erlangen-Nürnberg, Medical Informatics, Erlangen, Germany	Friedrich-Alexander-Universität Erlangen-Nürnberg, Medizinische Informatik, Erlangen, Deutschland
Jens	Pryzbilla		Clinical Trial Centre Leipzig (ZKS), Leipzig University, Leipzig, Germany	Zentrum für Klinische Studien, Universität Leipzig, Leipzig, Deutschland
Editha	Räuscher	0009-0008-5329-3819	TMF - Technology, Methods and Infrastructure for Networked Medical Research	TMF - Technologie und Methodenplattform für die vernetzte medizinische Forschung
Louisa	Redeker	0000-0003-4150-9084	Department of Clinical Pharmacology, School of Medicine, Faculty of Health, Witten/Herdecke University, Witten, Germany	Lehrstuhl für Klinische Pharmakologie, Department für Humanmedizin, Fakultät für Gesundheit, Universität Witten/Herdecke, Witten, Deutschland

Yvonne	Remane			Universitätsklinikum Leipzig
Andrea	Riedel	0009-0006-9389-9482	Erlangen University Hospital, Medical Center for Information and Communication Technology, Erlangen, Germany; Friedrich-Alexander-Universität Erlangen-Nürnberg, Medical Informatics, Erlangen, Germany	Universitätsklinikum Erlangen, Medizinisches Zentrum für Informations- und Kommunikationstechnologie, Erlangen, Deutschland; Friedrich-Alexander-Universität Erlangen-Nürnberg, Medizinische Informatik, Erlangen, Deutschland
Marietta	Rottenkolber			Ludwig-Maximilians-Universität München
Felix	Rottmann			UK Schleswig-Holstein, Campus Kiel
Friederike	Salman			Universitätsklinikum Hamburg-Eppendorf
Josef	Schepers			BIHealth | Charité Berlin
André	Scherag	0000-0002-9406-4704	Institute of Medical Statistics, Computer and Data Sciences (IMSID); Jena University Hospital; Jena; Germany	Institut für Medizinische Statistik, Informatik und Datenwissenschaften (IMSID); Universitätsklinikum Jena; Jena; Deutschland
Stefanie	Schild		Erlangen University Hospital, Medical Center for Information and Communication Technology, Erlangen, Germany	Universitätsklinikum Erlangen, Medizinisches Zentrum für Informations- und Kommunikationstechnologie, Erlangen, Deutschland
Florian	Schmidt	0000-0003-2027-8213	Institute of Medical informatics, Statistics and Epidemiology, University Leipzig, Leipzig, Germany	Universität Leipzig
Sven	Schmiedl	0000-0003-4844-0112	Helios University Clinic Wuppertal, Chair of Clinical Pharmacology, Faculty of Health, Department of Medicine, University of Witten/Herdecke, Germany	Helios Universitätsklinikum Wuppertal, Lehrstuhl für Klinische Pharmakologie, Fakultät für Gesundheit, Department Humanmedizin, Universität Witten/Herdecke
Katharina	Schmitz			Universitätsklinikum Aachen
Gerd	Schneider			Universitätsklinikum Heidelberg
Andreas	Scholtz			Universitätsklinikum Leipzig
Saskia	Schorn			Universitätsklinikum Aachen

Björn	Schreiweis	0000-0002-1748-1563	1: Institute for Medical Informatics and Statistics, Kiel University and University Hospital Schleswig-Holstein, Campus Kiel, Kiel, Germany; 2: Medical Data Integration Center, University Hospital Schleswig-Holstein, Germany	1: Institut für Medizinische Informatik und Statistik, Christian-Albrechts-Universität zu Kiel und Universitätsklinikum Schleswig-Holstein, Campus Kiel; 2: Medizinisches Datenintegrationszentrum, Universitätsklinikum Schleswig-Holstein
Susann	Schulze			Universitätsklinikum Hamburg-Eppendorf
Anna Kathrin	Schuster	0000-0002-5958-4560	Hospital Pharmacy, Jena University Hospital, Erlanger Allee 101, 07747 Jena, Thuringia, Germany	Apotheke des Universitätsklinikums Jena, Erlanger Allee 101, 07747 Jena, Thüringen, Germany
Matthias	Schwab		Dr. Margarete Fischer-Bosch Institute of Clinical Pharmacology, 70376 Stuttgart, Germany<br><br>Departments of Clinical Pharmacology, and of Pharmacy and Biochemistry, University Hospital Tuebingen, 72076 Tuebingen, Germany	Dr. Margarete Fischer-Bosch Institut für Klinische Pharmakologie, 70376 Stuttgart<br><br>Abteilung Klinische Pharmakologie Universitätsklinikum Tübingen, 72076 Tübingen
Hanna	Seidling	0000-0002-1215-634X	Heidelberg University, Medical Faculty Heidelberg / Heidelberg University Hospital, Internal Medicine IX - Department of Clinical Pharmacology and Pharmacoepidemiology, Cooperation Unit Clinical Pharmacy, Im Neuenheimer Feld 410, 69120 Heidelberg, Germany	Universitätsklinikum Heidelberg
Michael	Slupina			Universitätsklinikum Tübingen
Ronald	Speer			Universität Leipzig
Sebastian	Stäubert			Universität Leipzig

Daniel	Steinbach	0000-0003-2364-598X	1.) Institute of Laboratory Medicine, Clinical Chemistry and Molecular Diagnostics, University of Leipzig Medical Center, Leipzig, Germany<br>2.) Medical Informatics Center - Dept. for Clinical AI and Translational Medicine, University of Leipzig Medical Center	1.) Institut für Laboratoriumsmedizin, Klinische Chemie und Molekulare Diagnostik, Universitätsklinikum Leipzig, Leipzig, Deutschland<br>2.) Medizininformatikzentrum - Abteilung für medizinische KI und Translation, Universitätsklinikum Leipzig
Cornelia	Stelzer			Universitätsklinikum Leipzig
Holger	Stenzhorn	0000-0001-9744-174X	University Hospital Tübingen, Medical Data Integration Center, Tübingen, Germany; Saarland University, Faculty of Medicine, Data Integration Center, Homburg, Germany	Universitätsklinikum Tübingen, Medizinisches Datenintegrationszentrum, Tübingen, Deutschland; Universität des Saarlandes, Medizinische Fakultät, Datenintegrationszentrum, Homburg, Deutschland
Melanie	Straubmeier		Institute of Experimental and Clinical Pharmacology and Toxicology, Friedrich-Alexander-Universität Erlangen-Nürnberg, Erlangen, Germany	Institut für Experimentelle und Klinische Pharmakologie und Toxikologie, Friedrich-Alexander-Universität Erlangen-Nürnberg, Erlangen
Marcus	Strobel			Universitätsklinikum Leipzig
Alexander	Strübing	0000-0001-5151-7665	Institute for Medical Informatics, Statistics and Epidemiology (IMISE), Leipzig University, Leipzig, Germany	
Theresa	Terstegen	0009-0000-9689-6806	Heidelberg University, Medical Faculty Heidelberg / Heidelberg University Hospital, Internal Medicine IX - Department of Clinical Pharmacology and Pharmacoepidemiology, Cooperation Unit Clinical Pharmacy, Im Neuenheimer Feld 410, 69120 Heidelberg, Germany	Universitätsklinikum Heidelberg
Torsten	Thalheim	0000-0001-5489-7405		

Petra	Thürmann	0000-0001-9724-1422	Chair of Clinical Pharmacology, Faculty of Health, Department of Medicine, University of Witten/Herdecke	Helios Universitätsklinikum Wuppertal
Daniel	Tiller			Universitätsklinikum Halle
Patric	Tippmann	0000-0001-6521-8607	Institute of Medical Biometry and Statistics, Faculty of Medicine and Medical Center – University of Freiburg, Germany	Institut für Medizinische Biometrie und Statistik, Universitätsklinikum Freiburg, Medizinische Fakultät, Albert-Ludwigs-Universität Freiburg, Deutschland
Yeliz	Ucer			RWTH Aachen
Silvia	Unger			Universität Leipzig LIFE MC
Jan	Vogel			Universitätsklinikum Leipzig
Jonas	Wagner			Universität Leipzig LIFE MC
Julius	Wehrle			Universitätsklinikum Freiburg
Laura	Weisbach			Universitätsklinikum Jena
Sascha	Welten			RWTH Aachen
Thomas	Wendt	0009-0005-2287-9655	Data Integration Center, University of Leipzig Medical Center, Leipzig, Germany	Universitätsklinikum Leipzig
Anna Maria	Wermund	0000-0002-5735-0197	Department of Clinical Pharmacy, Institute of Pharmacy, University of Bonn, 53121 Bonn, Germany	Pharmazeutisches Institut, Abteilung Klinische Pharmazie, Universität Bonn
Reto	Wettstein			Universitätsklinikum Heidelberg
Ian	Wittenberg			Universitätsklinikum Halle
Maryam	Yahiaoui-Doktor			Universität Leipzig
Susanne	Zabka			Universitätsklinikum Freiburg
Sven	Zenker	0000-0003-0774-0725	Staff Unit for Medical and Scientific Technology Development and Coordination, University Hospital Bonn, Bonn, Germany; Applied Mathematical Physiology (AMP) Lab, Department of Anesthesiology and Intensive Care Medicine, University	Universitätsklinikum Bonn

			Hospital Bonn, Bonn, Germany; Applied Medical Informatics (AMI) Lab, Institute for Medical Biometry, Informatics and Epidemiology, University Hospital Bonn, Bonn, Germany	
Samira	Zeynalova			Universität Leipzig
Lukas	Zimmermann			Universitätsklinikum Tübingen
Daniela	Zöller			Universitätsklinikum Freiburg
