# Supplementary material for: Challenges in detecting and predicting adverse drug events via distributed analysis of electronic health record data from German university hospitals
Source: PLOS Digit Health. 2025 Jun 26;4(6):e0000892. doi: 10.1371/journal.pdig.0000892 (PMC12200832; doi:10.1371/journal.pdig.0000892)
Supplement: S2 File — (PDF) [file pdig.0000892.s002.pdf]

Supporting information file S2: Supplemental tables

Challenges in detecting and predicting adverse drug events via distributed analysis of electronic health record data from German university hospitals

Anna Maria Wermund[1], Torsten Thalheim[2,3,4], André Medek[5], Florian Schmidt[3], Thomas Peschel[3], Alexander Strübing[3], Daniel Neumann[3], André Scherag[6], Markus Loeffler[3], Miriam Kesselmeier[6,¶] and Ulrich Jaehde[1,¶*] for the POLAR_MI Consortium[+]

[+] The membership list of POLAR_MI is provided in Supporting information file S1 ("Membership list of POLAR_MI")

[1] Department of Clinical Pharmacy, Institute of Pharmacy, University of Bonn, Bonn, Germany.
[2] Interdisciplinary Centre for Bioinformatics, Leipzig University, Leipzig, Germany
[3] Institute for Medical Informatics, Statistics and Epidemiology (IMISE), Leipzig University, Leipzig, Germany
[4] Deutsches Biomasseforschungszentrum gGmbH, Torgauer Str. 116, 04347 Leipzig, Germany
[5] Medical & Scientific Technology Development & Coordination (MWTek), University Hospital Bonn, 53127 Bonn, Germany
[6] Institute of Medical Statistics, Computer and Data Sciences (IMSID), Jena University Hospital – Friedrich Schiller University Jena, Jena, Germany

[¶] Equal contribution

* u.jaehde@uni-bonn.de

List of tables

Table A in S2 Supplemental tables. Definitions and assumptions for outcomes and other variables 3

Table B in S2 Supplemental tables. Additional information for laboratory value definitions 12

Table C in S2 Supplemental tables. Meta-analysed description of the missing data pattern for the analysis (B1.a) of the outcome GI bleeding 17

Table D in S2 Supplemental tables. Meta-analysed description of the missing data pattern for the analysis (B1.b) of the outcome GI bleeding 19

Table E in S2 Supplemental tables. Meta-analysed description of the missing data pattern for the analysis (B1.c) of the outcome GI bleeding 21

Table F in S2 Supplemental tables. Meta-analysed description of the missing data pattern for the analysis (H1.a) of the outcome drug-related hypoglycaemia 24

Table G in S2 Supplemental tables. Meta-analysed description of the missing data pattern for the analysis (H1.b) of the outcome drug-related hypoglycaemia 25

Table H in S2 Supplemental tables. Meta-analysed description of the missing data pattern for the analysis (H1.c) of the outcome drug-related hypoglycaemia 26

Table I in S2 Supplemental tables. Meta-analysed description of the study population for the analysis (B1.a) of the outcome GI bleeding 27

Table J in S2 Supplemental tables. Meta-analysed description of the study population for the analysis (B1.b) of the outcome GI bleeding 28

Table K in S2 Supplemental tables. Meta-analysed description of the study population for the analysis (B1.c) of the outcome GI bleeding 29

Table L in S2 Supplemental tables. Meta-analysed description of the study population for the analysis (H1.a) of the outcome drug-related hypoglycaemia 30

Table M in S2 Supplemental tables. Meta-analysed description of the study population for the analysis (H1.b) of the outcome drug-related hypoglycaemia 31

Table N in S2 Supplemental tables. Meta-analysed description of the study population for the analysis (H1.c) of the outcome drug-related hypoglycaemia 32

Table O in S2 Supplemental tables. Meta-analysed results from the regression modelling for the analysis (B1.a) of the outcome GI bleeding 33

Table P in S2 Supplemental tables. Meta-analysed results from the regression modelling for the analysis (B1.b) of the outcome GI bleeding 34

Table Q in S2 Supplemental tables. Meta-analysed results from the regression modelling for the analysis (H1.a) of the outcome drug-related hypoglycaemia 36

Table R in S2 Supplemental tables. Meta-analysed results from the regression modelling for the analysis (H1.b) of the outcome drug-related hypoglycaemia 37

Table A in S2 Supplemental tables. Definitions and assumptions for outcomes and other variables. For the definitions, the German Modification of the International Statistical Classification of Diseases and Related Health Problems (10th Revision, ICD-10-GM) was used for diagnoses, the Anatomical Therapeutic Chemical (ATC) classification system for drugs and the Logical Observation Identifiers Names and Codes (LOINC) for laboratory values. An asterisk (*) indicates that all codes beginning with that prefix have been used. Besides definitions, we provide the scale (nominal, ordinal, metric) as well as the related coding for categorical variables, reasons for missing information on the respective variable, the underlying core data set (CDS) resource and additional comments, if any. For variables related to medications, diagnoses and laboratory values, the time point or period was only applied in those analyses considering the chronology of events. For further details, we refer to Kesselmeier et al. (submitted to *PLoS Digital Health*, PDIG-D-25-00026). Further abbreviations: ∈, element of; ALT, alanine transaminase; ASA, acetylsalicylic acid; AST, aspartate aminotransferase; DM, diabetes mellitus; GI, gastrointestinal; NA, not available; NSAID, non-steroidal anti-inflammatory drug; SSRI, selective serotonin reuptake inhibitor.

Variable	Definition and further information
Outcomes	
Bleeding and perforation of the upper gastrointestinal tract; referred to as "GI bleeding"	• Definition: [ICD-10-GM-Code] ∈ {K22.81, K22.3, K25.0, K25.1, K25.2, K25.4, K25.5, K25.6, K26.0, K26.1, K26.2, K26.4, K26.5, K26.6, K27.0, K27.1, K27.2, K27.4, K27.5, K27.6, K29.0, K92.0, K92.1, K92.2}<br>• Scale: nominal (binary)<br>• Value coding: 0 = no GI bleeding documented; 1 = GI bleeding documented<br>• Time point / period of time (if required): during hospital stay (between day 2 and day of discharge; calendar day, not 24 hours)<br>• Missing value: information on diagnoses not available (not assessed or no ICD-10-GM-Code documented at all or not readable in the data)<br>• CDS resource: Condition<br>• Comment: When considering time points, encounters were excluded if they had a coded GI bleeding on the day of admission, as it can be assumed that if a GI bleeding recured during hospitalisation, it was the same GI bleeding that was present on admission and therefore triggered by outpatient medication.
Hypoglycaemia	• Definition: [LOINC-Code] ∈ {15074-8, 14749-6, 2345-7, 2339-0, 14743-9, 39480-9, 41653-7, 32016-8, 41651-1, 41652-9, 100746-7, 39481-7, 51596-5, 74774-1, 77135-2, 72516-8, 2340-8, 2341-6, 35211-2, 6777-7}<br>• Scale: nominal (binary)<br>• Value coding: 0 = no hypoglycaemia documented; 1 = hypoglycaemia documented<br>• Time point / period of time (if required): during hospital stay (between day 2 and day of discharge; calendar day, not 24 hours)

	• Missing value: LOINC codes not available (laboratory value not obtained or not documented with the appropriate LOINC code during the stay or LOINC code value not provided/readable in the data (including deviation from expected units)<br>• CDS resource: Observation<br>• Comment: We did not exclude encounters with a laboratory value indicating hypoglycaemia on the day of admission, as hypoglycaemia can be treated and resolved more quickly than GI bleeding, and therefore a new episode of hypoglycaemia may occur during hospitalisation. If several laboratory results were available, the most severe value (during the specified period of time) was used. For further details see Table B in S2 Supplemental tables.
Outcome-related inclusion criterion	
Antihyperglycaemic drug	• Definition: [ATC-Code] ∈ {A10*, A08AX02}<br>• Scale: nominal (binary)<br>• Value coding: 0 = no antihyperglycemic drug documented; 1 = antihyperglycemic drug documented<br>• Time point / period of time (if required): day of hospital admission (day 1; calendar day, not 24 hours)<br>• Missing value: information on medication not available (no medication intake/prescription or no ATC Code documented at all or not readable in the data)<br>• CDS resource: Medication and (MedicationAdministration or MedicationStatement). MedicationAdministration and MedicationStatement contain the administration/prescription with a reference to the medication in the CDS resource Medication.<br>• Comment: none
Covariates for regression models	
Age, in years	• Definition: building ([day of hospitalisation] - [birthday]) and rounding down to year<br>• Scale: metric (integer)<br>• Value coding: none<br>• Time point / period of time (if required): day of hospital admission (day 1; calendar day, not 24 hours)<br>• Missing value: day of hospitalisation or birthday missing<br>• CDS resource: Encounter (day of hospitalization = Encounter.period.start), Patient (birthday = Patient.birthDate)<br>• Comment: If the exact day or month of birth was missing, the first day of the month or January was used (e.g. for August 1999, 1 August 1999 was used and for 1999, 1 January 1999 was used).

Gender	• Definition: not applicable<br>• Scale: nominal<br>• Value coding: 0 = female; 1 = male<br>• Time point / period of time (if required): day of hospital admission (day 1; calendar day, not 24 hours)<br>• Missing value: information missing<br>• CDS resource: Patient<br>• Comment: The local retrieval and analysis scripts were programmed to deal with gender diverse, but this gender was not coded in the analysed data.
NSAID	• Definition: [ATC-Code] ∈ {M01A*, M01BA01, R05XA10, M01BA04, M01BA05, M01BA08, C01EB03, N02AJ05, N02AJ08, N02AJ19, R01BA57, C01EB16, N02AJ14, C08CA51, L01XX33, N02AJ16, M01BA03, N02AJ02, N02AJ07, N02AJ18, N02BA01, N02BA51, N02BA71, R05XA02, R05XA22}<br>• Scale: nominal (binary)<br>• Value coding: 0 = no NSAID documented; 1 = NSAID documented<br>• Time point / period of time (if required): day of hospital admission (day 1; calendar day, not 24 hours)<br>• Missing value: information on medication not available (no medication intake/prescription or no ATC Code documented at all or not readable in the data)<br>• CDS resource: Medication and (MedicationAdministration or MedicationStatement). MedicationAdministration and MedicationStatement contain the administration/prescription with a reference to the medication in the CDS resource Medication.<br>• Comment: none
ASA	• Definition: [ATC-Code] ∈ {B01AC06, B01AC34, B01AC36, B01AC56, B01AC86, C07FX02, C07FX03, C07FX04, C10BX01, C10BX02, C10BX04, C10BX05, C10BX06, C10BX08, C10BX12, M01BA03, N02AJ02, N02AJ07, N02AJ18, N02BA01, N02BA51, N02BA71, R05XA02, R05XA22}<br>• Scale: nominal (binary)<br>• Value coding: 0 = no ASA documented; 1 = ASA documented<br>• Time point / period of time (if required): day of hospital admission (day 1; calendar day, not 24 hours)<br>• Missing value: information on medication not available (no medication intake/prescription or no ATC Code documented at all or not readable in the data)<br>• CDS resource: Medication and (MedicationAdministration or MedicationStatement). MedicationAdministration and

	MedicationStatement contain the administration/prescription with a reference to the medication in the CDS resource Medication.<br>• Comment: none
SSRI	• Definition: [ATC-Code] ∈ {N06AB*, N06CA03}<br>• Scale: nominal (binary)<br>• Value coding: 0 = no SSRI documented; 1 = SSRI documented<br>• Time point / period of time (if required): day of hospital admission (day 1; calendar day, not 24 hours)<br>• Missing value: information on medication not available (no medication intake/prescription or no ATC Code documented at all or not readable in the data)<br>• CDS resource: Medication and (MedicationAdministration or MedicationStatement). MedicationAdministration and MedicationStatement contain the administration/prescription with a reference to the medication in the CDS resource Medication.<br>• Comment: none
Bisphosphonate	• Definition: [ATC-Code] ∈ {M05BA*, M05BB*}<br>• Scale: nominal (binary)<br>• Value coding: 0 = no bisphosphonate documented; 1 = bisphosphonate documented<br>• Time point / period of time (if required): day of hospital admission (day 1; calendar day, not 24 hours)<br>• Missing value: information on medication not available (no medication intake/prescription or no ATC Code documented at all or not readable in the data)<br>• CDS resource: Medication and (MedicationAdministration or MedicationStatement). MedicationAdministration and MedicationStatement contain the administration/prescription with a reference to the medication in the CDS resource Medication.<br>• Comment: none
Any insulin	• Definition: [ATC-Code] ∈ {A10A*}<br>• Scale: nominal (binary)<br>• Value coding: 0 = no insulin documented; 1 = insulin documented<br>• Time point / period of time (if required): day of hospital admission (day 1; calendar day, not 24 hours)<br>• Missing value: information on medication not available (no medication intake/prescription or no ATC Code

	documented at all or not readable in the data)<br>• CDS resource: Medication and (MedicationAdministration or MedicationStatement). MedicationAdministration and MedicationStatement contain the administration/prescription with a reference to the medication in the CDS resource Medication.<br>• Comment: none
Long-acting insulin	• Definition: [ATC-Code] ∈ {A10AE*, A10AD06}<br>• Scale: nominal (binary)<br>• Value coding: 0 = no long-acting insulin documented; 1 = long-acting insulin documented<br>• Time point / period of time (if required): day of hospital admission (day 1; calendar day, not 24 hours)<br>• Missing value: information on medication not available (no medication intake/prescription or no ATC Code documented at all or not readable in the data)<br>• CDS resource: Medication and (MedicationAdministration or MedicationStatement). MedicationAdministration and MedicationStatement contain the administration/prescription with a reference to the medication in the CDS resource Medication.<br>• Comment: none
Liver disease	• Definition: [ICD-10-GM-Code] ∈ {R16.0, R16.2, C22*, Q44.6, Q44.7, C78.7, A06.4*, B67.0, B67.5, B67.8, D13.4, S36.10, S36.11, S36.12, S36.13, S36.14, S36.15, S36.16, T86.4*, Z75.67, Z75.77, B25.1*, B58.1*, B15*, B16*, B17*, B18*, B19*, I82.0, K70*, K71*, K72*, K73*, K74*, K75*, K76*, K77*, Z94.4}<br>• Scale: nominal (binary)<br>• Value coding: 0 = no liver disease documented; 1 = liver disease documented<br>• Time point / period of time (if required): day of hospital admission (day 1; calendar day, not 24 hours)<br>• Missing value: information on diagnoses not available (not assessed or no ICD-10-GM-Code documented at all or not readable in the data)<br>• CDS resource: Condition<br>• Comment: none
Heart failure	• Definition: [ICD-10-GM-Code] ∈ {I50*, I13.0*, I13.2*, I11.0*}<br>• Scale: nominal (binary)<br>• Value coding: 0 = no heart failure documented; 1 = heart failure documented

	• Time point / period of time (if required): day of hospital admission (day 1; calendar day, not 24 hours)<br>• Missing value: information on diagnoses not available (not assessed or no ICD-10-GM-Code documented at all or not readable in the data)<br>• CDS resource: Condition<br>• Comment: none
Type of diabetes mellitus (DM)	• Definition:<br>◦ Diabetes mellitus type 1: [ICD-10-GM-Code] ∈ {E10*}<br>◦ Diabetes mellitus type 2: [ICD-10-GM-Code] ∈ {E11*}<br>◦ Diabetes mellitus other or unspecified type: [ICD-10-GM-Code] ∈ {E12*, E13*, E14*}<br>• Scale: nominal<br>• Value coding: 0 = no DM documented; 1 = type 1 DM documented; 2 = type 2 DM documented; 3 = other or unspecified type of DM documented<br>• Time point / period of time (if required): day of hospital admission (day 1; calendar day, not 24 hours)<br>• Missing value: information on diagnoses not available (not assessed or no ICD-10-GM-Code documented at all or not readable in the data)<br>• CDS resource: Condition<br>• Comment: We aimed at assessing the impact of DM type 1 and DM type 2 on the outcome. Therefore, we were not primarily interested in whether there was a co-occurrence of DM type 1 or 2 with “other or unspecified” type of DM. Furthermore, we did not initially expect the co-occurrence of DM type 1 and DM type 2, so one variable was created to gather the desired information. However, we observed the co-occurrence of different types of DM, so the following rules applied when encounters were diagnosed with more than one type of DM:<br>◦ If a type 1 DM and a type 2 DM were documented, the variable was set to unknown (NA). Thus, these encounters were excluded from modelling, but the number of affected encounters (and patients) was assessed.<br>◦ If a type 1 DM and an “other or unspecified type” of DM were documented, the variable was set to type 1 DM.<br>◦ If a type 2 DM and an ”other or unspecified type” of diabetes mellitus were documented, the variable was set to type 2 DM.
AST increased	• Definition: [LOINC-Code] ∈ {1920-8, 88112-8, 30239-8, 48136-6}<br>• Scale: nominal (binary)<br>• Value coding: 0 = no increased AST documented; 1 = increased AST documented

	• Time point / period of time (if required): day of hospital admission (day 1; calendar day, not 24 hours)<br>• Missing value: LOINC codes not available (laboratory value not obtained or not documented with the appropriate LOINC code during the stay or LOINC code value not provided/readable in the data (including deviation from expected units)<br>• CDS resource: Observation<br>• Comment: If several laboratory results were available, the most severe value (during the specified period of time) was used. For further details see Table B in S2 Supplemental tables.
ALT increased	• Definition: [LOINC-Code] ∈ {76625-3, 1742-6, 1743-4, 1744-2, 48134-1, 77144-4, 76625-3}<br>• Scale: nominal (binary)<br>• Value coding: 0 = no increased ALT documented; 1 = increased ALT documented<br>• Time point / period of time (if required): day of hospital admission (day 1; calendar day, not 24 hours)<br>• Missing value: LOINC codes not available (laboratory value not obtained or not documented with the appropriate LOINC code during the stay or LOINC code value not provided/readable in the data (including deviation from expected units)<br>• CDS resource: Observation<br>• Comment: If several laboratory results were available, the most severe value (during the specified period of time) was used. For further details see Table B in S2 Supplemental tables.
Serum albumin categorised	• Definition: [LOINC-Code] ∈ {77148-5, 61151-7, 61152-5, 54347-0, 62234-0, 62235-7, 76631-1, 1751-7, 2862-1, 101198-0}<br>• Scale: ordinal (treated as categorical variable)<br>• Value coding: 0 = normal serum albumin documented; 1 = decreased serum albumin documented; 2 = increased serum albumin documented<br>• Time point / period of time (if required): day of hospital admission (day 1; calendar day, not 24 hours)<br>• Missing value: LOINC codes not available (laboratory value not obtained or not documented with the appropriate LOINC code during the stay or LOINC code value not provided/readable in the data (including deviation from expected units)<br>• CDS resource: Observation<br>• Comment: If several laboratory results were available, the most severe value (during the specified period of time) was used. This variable was set to unknown (NA) for encounters with conflicting information (one value increased

	and another decreased on the day of interest or over the whole hospital stay). Within risk modelling, increased serum albumin and normal serum albumin were combined into one category (value coding: 0), i.e. the independent variable assessed the impact of decreased serum albumin compared to non-decreased, because increased serum albumin was rarely detected and, thus, hindered (numerically stable) estimation within regression modelling. For further details see Table B in S2 Supplemental tables.
Haemoglobin categorised	• Definition: [LOINC-Code] ∈ {20509-6, 718-7, 30350-3, 59260-0, 14775-1, 30313-1, 97550-8, 97555-7, 93846-4, 76769-9, 75928-2, 55782-7, 97556-5}<br>• Scale: ordinal (treated as categorical variable)<br>• Value coding: 0 = normal haemoglobin documented; 1 = decreased haemoglobin documented; 2 = increased haemoglobin documented<br>• Time point / period of time (if required): day of hospital admission (day 1; calendar day, not 24 hours)<br>• Missing value: LOINC codes not available (laboratory value not obtained or not documented with the appropriate LOINC code during the stay or LOINC code value not provided/readable in the data (including deviation from expected units)<br>• CDS resource: Observation<br>• Comment: If several laboratory results were available, the most severe value (during the specified period of time) was used. This variable was set to unknown (NA) for encounters with conflicting information (one value increased and another decreased on the day of interest or over the whole hospital stay). Within risk modelling, increased haemoglobin and normal haemoglobin were combined into one category (value coding: 0), i.e. the independent variable assessed the impact of decreased haemoglobin compared to non-decreased, because increased haemoglobin was rarely detected and, thus, hindered (numerically stable) estimation within regression modelling. For further details see Table B in S2 Supplemental tables.
Creatinine	• Definition: [LOINC-Code] ∈ {59826-8, 14682-9, 2160-0, 21232-4, 38483-4, 77140-2, 59826-8, 44784-7, 101475-2, 35203-9}<br>• Scale: metric<br>• Value coding: none<br>• Time point / period of time (if required): day of hospital admission (day 1; calendar day, not 24 hours)<br>• Missing value: LOINC codes not available (laboratory value not obtained or not documented with the appropriate LOINC code during the stay or LOINC code value not provided/readable in the data (including deviation from

	expected units)<br>• <u>CDS resource:</u> Observation<br>• <u>Comment:</u> If several laboratory results were available, the most severe value (during the specified period of time) was used. For further details see Table B in S2 Supplemental tables.

Table B in S2 Supplemental tables. Additional information for laboratory value definitions. Due to the heterogeneity in the presentation of laboratory values across centres, it was necessary to identify possible units, including associated cut-off values and plausible value ranges. In some cases, conversion factors were established. Due to the heterogeneity in required decimal places, we did not unify the notation in terms of the number of decimal places provided.

Measure-related unit	Gender	Normal range		Plausibility range		Conversion factor
		Lower limit	Upper limit	Lower limit	Upper limit	
Hypoglycaemia						
mmol/L	male	3	Infinite	0.3	55.5	-
mg/dl	male	55	Infinite	5	1,000	-
mmol/L	female	3	Infinite	0.3	55.5	-
mg/dl	female	55	Infinite	5	1,000	-
AST and ALT						
U/L	male	10	50	0	Infinite	-
IU/L	male	10	50	0	Infinite	-
µmol/(min*L)	male	10	50	0	Infinite	-
µmol/(h*L)	male	600.0002	3,000.0012	0	Infinite	-
µmol/(h*mL)	male	0.5999	2.994	0	Infinite	-
umol/(min*L)	male	10	50	0	Infinite	-
umol/(h*L)	male	600.0002	3,000.0012	0	Infinite	-
umol/(h*mL)	male	0.5999	2.994	0	Infinite	-
µmol/(L*min)	male	10	50	0	Infinite	-
µmol/(L*h)	male	600.0002	3,000.0012	0	Infinite	-
µmol/(mL*h)	male	0.5999	2.994	0	Infinite	-
umol/(L*min)	male	10	50	0	Infinite	-
umol/(L*h)	male	600.0002	3,000.0012	0	Infinite	-
umol/(mL*h)	male	0.5999	2.994	0	Infinite	-
µmol/min/L	male	10	50	0	Infinite	-
µmol/h/L	male	600.0002	3,000.0012	0	Infinite	-
µmol/h/mL	male	0.5999	2.994	0	Infinite	-
umol/min/L	male	10	50	0	Infinite	-

umol/h/L	male	600.0002	3,000.0012	0	Infinite	-
umol/h/mL	male	0.5999	2.994	0	Infinite	-
µmol/L/min	male	10	50	0	Infinite	-
µmol/L/h	male	600.0002	3,000.0012	0	Infinite	-
µmol/mL/h	male	0.5999	2.994	0	Infinite	-
umol/L/min	male	10	50	0	Infinite	-
umol/L/h	male	600.0002	3,000.0012	0	Infinite	-
umol/mL/h	male	0.5999	2.994	0	Infinite	-
nkat/L	male	166.667	833.3333	0	Infinite	-
µkat/L	male	0.1667	0.8333	0	Infinite	-
ukat/L	male	0.1667	0.8333	0	Infinite	-
nmol/(s*L)	male	166.6667	833.3333	0	Infinite	-
µmol/(s*L)	male	0.1667	0.8333	0	Infinite	-
umol/(s*L)	male	0.1667	0.8333	0	Infinite	-
nmol/(L*s)	male	166.6667	833.3333	0	Infinite	-
µmol/(L*s)	male	0.1667	0.8333	0	Infinite	-
umol/(L*s)	male	0.1667	0.8333	0	Infinite	-
nmol/s/L	male	166.6667	833.3333	0	Infinite	-
µmol/s/L	male	0.1667	0.8333	0	Infinite	-
umol/s/L	male	0.1667	0.8333	0	Infinite	-
nmol/L/s	male	166.6667	833.3333	0	Infinite	-
µmol/L/s	male	0.1667	0.8333	0	Infinite	-
umol/L/s	male	0.1667	0.8333	0	Infinite	-
U/L	female	10	35	0	Infinite	-
IU/L	female	10	35	0	Infinite	-
µmol/(min*L)	female	10	35	0	Infinite	-
µmol/(h*L)	female	600.0002	2,100.0008	0	Infinite	-
µmol/(h*mL)	female	0.5999	2.0996	0	Infinite	-
umol/(min*L)	female	10	35	0	Infinite	-
umol/(h*L)	female	600.0002	2,100.0008	0	Infinite	-

umol/(h*mL)	female	0.5999	2.0996	0	Infinite	-
µmol/(L*min)	female	10	35	0	Infinite	-
µmol/(L*h)	female	600.0002	2,100.0008	0	Infinite	-
µmol/(mL*h)	female	0.5999	2.0996	0	Infinite	-
umol/(L*min)	female	10	35	0	Infinite	-
umol/(L*h)	female	600.0002	2,100.0008	0	Infinite	-
umol/(mL*h)	female	0.5999	2.0996	0	Infinite	-
µmol/min/L	female	10	35	0	Infinite	-
µmol/h/L	female	600.0002	2,100.0008	0	Infinite	-
µmol/h/mL	female	0.5999	2.0996	0	Infinite	-
umol/min/L	female	10	35	0	Infinite	-
umol/h/L	female	600.0002	2,100.0008	0	Infinite	-
umol/h/mL	female	0.5999	2.0996	0	Infinite	-
µmol/L/min	female	10	35	0	Infinite	-
µmol/L/h	female	600.0002	2,100.0008	0	Infinite	-
µmol/mL/h	female	0.5999	2.0996	0	Infinite	-
umol/L/min	female	10	35	0	Infinite	-
umol/L/h	female	600.0002	2,100.0008	0	Infinite	-
umol/mL/h	female	0.5999	2.0996	0	Infinite	-
nkat/L	female	166.667	583.3333	0	Infinite	-
µkat/L	female	0.1667	0.5833	0	Infinite	-
ukat/L	female	0.1667	0.5833	0	Infinite	-
nmol/(s*L)	female	166.6667	583.3333	0	Infinite	-
µmol/(s*L)	female	0.1667	0.5833	0	Infinite	-
umol/(s*L)	female	0.1667	0.5833	0	Infinite	-
nmol/(L*s)	female	166.6667	583.3333	0	Infinite	-
µmol/(L*s)	female	0.1667	0.5833	0	Infinite	-
umol/(L*s)	female	0.1667	0.5833	0	Infinite	-
nmol/s/L	female	166.6667	583.3333	0	Infinite	-
µmol/s/L	female	0.1667	0.5833	0	Infinite	-

umol/s/L	female	0.1667	0.5833	0	Infinite	-
nmol/L/s	female	166.6667	583.3333	0	Infinite	-
µmol/L/s	female	0.1667	0.5833	0	Infinite	-
umol/L/s	female	0.1667	0.5833	0	Infinite	-
Serum albumin						
g/L	male	35	54	10	100	-
g/dL	male	3.5	5.4	1	10	-
g/100mL	male	3.5	5.4	1	10	-
g%	male	3.5	5.4	1	10	-
mg/ml	male	35	54	10	100	-
mmol/L	male	0.5073	0.7826	0.14493	1,449.3	-
µmol/L	male	507.255	782.622	144.93	1,449.3	-
umol/L	male	507.255	782.622	144.93	1,449.3	-
g/L	female	35	54	10	100	-
g/dL	female	3.5	5.4	1	10	-
g/100mL	female	3.5	5.4	1	10	-
g%	female	3.5	5.4	1	10	-
mg/ml	female	35	54	10	100	-
mmol/L	female	0.5073	0.7826	0.14493	1,449.3	-
µmol/L	female	507.255	782.622	144.93	1,449.3	-
umol/L	female	507.255	782.622	144.93	1,449.3	-
Creatinine						
mg/dL	male	-	-	0	Infinite	0.011
mg/100mL	male	-	-	0	Infinite	0.011
mg%	male	-	-	0	Infinite	0.011
mg/L	male	-	-	0	Infinite	0.113
µg/mL	male	-	-	0	Infinite	0.113
ug/mL	male	-	-	0	Infinite	0.113
mmol/L	male	-	-	0	Infinite	0.001
µmol/L	male	-	-	0	Infinite	1

umol/L	male	-	-	0	Infinite	1
mg/dL	female	-	-	0	Infinite	0.011
mg/100mL	female	-	-	0	Infinite	0.011
mg%	female	-	-	0	Infinite	0.011
mg/L	female	-	-	0	Infinite	0.113
µg/mL	female	-	-	0	Infinite	0.113
ug/mL	female	-	-	0	Infinite	0.113
mmol/L	female	-	-	0	Infinite	0.001
µmol/L	female	-	-	0	Infinite	1
umol/L	female	-	-	0	Infinite	1
Haemoglobin						
g/dL	male	13	18	1.6	48.3	-
g/100mL	male	13	18	1.6	48.3	-
G%	male	13	18	1.6	48.3	-
mg/mL	male	130	180	16	483	-
g/L	male	130	180	16	483	-
mmol/L	male	8.07	11.17	1	30	-
g/dL	female	12	16	1.6	48.3	-
g/100mL	female	12	16	1.6	48.3	-
G%	female	12	16	1.6	48.3	-
mg/mL	female	120	160	16	483	-
g/L	female	120	160	16	483	-
mmol/L	female	7.44	9.92	1	30	-

Table C in S2 Supplemental tables. Meta-analysed description of the missing data pattern for the analysis (B1.a) of the outcome GI bleeding across seven centres. The patterns are defined by the outcome (dependent variable) and the related independent variables included in the regression models. Each row corresponds to a specific pattern, with green indicating no missing information and red indicating missing information. Abbreviations: ALT, alanine transaminase; ASA, acetylsalicylic acid; AST, aspartate aminotransferase; GI, gastrointestinal; ID, identifier; NSAID, non-steroidal anti-inflammatory drug; SSRI, selective serotonin reuptake inhibitor.

	Outcome	Independent variables												Per pattern: number of …	
Pattern ID	GI bleeding	Age	Gender	NSAID	ASA	SSRI	Bisphosphonate	Liver disease	AST increased	ALT increased	Serum albumin decreased	Haemoglobin decreased	Creatinine	… missing values	… encounters
1														0	66,038
2														1	8,308
3														1	2,502
4														2	85
5														1	58,348
6														2	7,817
7														2	4,290
8														3	228
9														1	4,486
10														2	213
11														2	91
12														3	1
13														2	6,589
14														3	1,307
15														3	175
16														4	24
17														1	4,532
18														2	378
19														2	47
20														3	3
21														2	17,880
22														3	1,193
23														3	720
24														4	25
25														2	2,548
26														3	325
27														3	393
28														4	88
29														3	29,131
30														4	29,965
31														4	4,175
32														5	78,441
33														4	3
34														5	13
35														6	2

36														8	3
37														9	1
38														5	14
39														6	4
40														2	1,127
41														3	9
42														3	236
43														4	1
44														3	119
45														4	14
46														4	5
47														4	1
48														5	1
49														4	3
50														3	26
51														5	1
52														4	94
53														5	1
54														6	1
55														4	6
56														6	7
57														5	235
58														6	134
59														6	46
60														7	3,549
Per variable: number of encounters with missing information															
Frequency	5,616	0	18	22	22	22	22	5,616	173,966	161,956	244,546	95,156	132,128		
Variable	GI bleeding	Age	Gender	NSAID	ASA	SSRI	Bisphosphonate	Liver disease	AST increased	ALT increased	Serum albumin	Haemoglobin	Creatinine		

Table D in S2 Supplemental tables. Meta-analysed description of the missing data pattern for the analysis (B1.b) of the outcome GI bleeding across five centres. The patterns are defined by the outcome (dependent variable) and the related independent variables included in the regression models. Each row corresponds to a specific pattern, with green indicating no missing information and red indicating missing information. Abbreviations: ALT, alanine transaminase; ASA, acetylsalicylic acid; AST, aspartate aminotransferase; GI, gastrointestinal; ID, identifier; NSAID, non-steroidal anti-inflammatory drug; SSRI, selective serotonin reuptake inhibitor.

	Outcome	Independent variables											Per pattern: number of …	
Pattern ID	GI bleeding	Age	Gender	NSAID	ASA	SSRI	Bisphosphonate	Liver disease	AST increased	ALT increased	Serum albumin decreased	Haemoglobin decreased	Creatinine	… missing values	… encounters
1														0	61,317
2														1	8
3														1	1,744
4														2	11
5														1	49,678
6														2	235
7														2	1,100
8														3	26
9														1	4,240
10														2	4
11														2	14
12														3	1
13														2	5,927
14														3	14
15														3	29
16														4	5
17														1	4,411
18														2	1
19														2	32
20														3	1
21														2	16,859
22														3	11
23														3	69
24														4	4
25														2	2,265
26														3	33
27														3	295
28														4	82
29														3	21,182
30														4	8,077
31														4	1,536
32														5	57,992
33														5	14
34														6	3
35														2	1,126

36														3	234
37														3	119
38														4	5
39														5	1
40														4	3
41														3	26
42														5	1
43														4	93
44														6	1
45														4	6
46														6	7
47														5	233
48														6	120
49														6	46
50														7	3,514
Per variable: number of encounters with missing information															
Frequency	5,535	0	17	0	0	0	0	5,535	116,914	105,643	166,895	66,767	70,152		
Variable	GI bleeding	Age	Gender	NSAID	ASA	SSRI	Bisphosphonate	Liver disease	AST increased	ALT increased	Serum albumin decreased	Haemoglobin decreased	Creatinine		

Table E in S2 Supplemental tables. Meta-analysed description of the missing data pattern for the analysis (B1.c) of the outcome GI bleeding across seven centres, which were also included in the analysis (B1.a). The patterns are defined by the outcome (dependent variable) and the related independent variables included in the regression models. Each row corresponds to a specific pattern, with green indicating no missing information and red indicating missing information. Abbreviations: ALT, alanine transaminase; ASA, acetylsalicylic acid; AST, aspartate aminotransferase; GI, gastrointestinal; ID, identifier; NSAID, non-steroidal anti-inflammatory drug; SSRI, selective serotonin reuptake inhibitor.

	Outcome	Independent variables												Per pattern: number of …	
Pattern ID	GI bleeding	Age	Gender	NSAID	ASA	SSRI	Bisphosphonate	Liver disease	AST increased	ALT increased	Serum albumin decreased	Haemoglobin decreased	Creatinine	… missing values	… encounters
1														0	31,034
2														1	3,335
3														1	3,896
4														2	63
5														1	37,287
6														2	5,437
7														2	2,983
8														3	154
9														1	3,684
10														2	69
11														2	228
12														3	14
13														2	3,549
14														3	2,793
15														3	113
16														4	73
17														1	1,482
18														2	307
19														2	17
20														3	7
21														2	13,444
22														3	1,842
23														3	681
24														4	48
25														2	1,003
26														3	78
27														3	149
28														4	89
29														3	16,073
30														4	23,920
31														4	2,779
32														5	171,390
33														4	3
34														5	8

35														6	4
36														8	3
37														9	4
38														5	10
39														6	8
40														1	379
41														2	39
42														2	6
43														3	1
44														2	392
45														3	31
46														3	21
47														4	1
48														2	79
49														3	2
50														3	36
51														4	12
52														2	20
53														3	4
54														3	247
55														4	171
56														4	1
57														5	1
58														3	7
59														4	1
60														4	101
61														5	265
62														5	5
63														6	503
64														2	338
65														3	5
66														3	185
67														4	2
68														3	47
69														4	11
70														4	4
71														3	1
72														4	1
73														5	1
74														4	30
75														5	4
76														6	2
77														4	2
78														5	1
79														5	1
80														6	3
81														5	271
82														6	137
83														6	112
84														7	4,458
Per variable: number of encounters with missing information															

Variable	Frequency
GI bleeding	7,941
Age	0
Gender	18
NSAID	22
ASA	22
SSRI	22
Bisphosphonate	22
Liver disease	5,616
AST increased	239,681
ALT increased	232,028
Serum albumin decreased	289,468
Haemoglobin decreased	188,016
Creatinine	215,289

Table F in S2 Supplemental tables. Meta-analysed description of the missing data pattern for the analysis (H1.a) of the outcome drug-related hypoglycaemia across six centres. The patterns are defined by the outcome (dependent variable) and the related independent variables included in the regression models. Each row corresponds to a specific pattern, with green indicating no missing information and red indicating missing information. Abbreviation: ID, identifier.

	Outcome	Independent variables								Per pattern: number of …	
Pattern ID	Drug-related hypoglycaemia	Age	Gender	Any insulin	Long-acting insulin	Heart failure	Diabetes mellitus type	Serum albumin decreased	Creatinine	… missing values	… encounters
1										0	14,108
2										1	25
3										1	16,067
4										2	2,387
5										1	55
6										2	34
7										3	1
8										2	267
9										3	40
10										4	7
11										1	909
12										2	3
13										2	1,696
14										3	7,833
15										2	5
16										3	2
17										4	21
18										4	3
19										4	42
20										5	594
21										3	2
Per variable: number of encounters with missing information											
Frequency	11,110	0	2	0	0	953	1,071	28,726	10,874		
Variable	Drug-related hypoglycaemia	Age	Gender	Any insulin	Long-acting insulin	Heart failure	Diabetes mellitus type	Serum albumin decreased	Creatinine		

Table G in S2 Supplemental tables. Meta-analysed description of the missing data pattern for the analysis (H1.b) of the outcome drug-related hypoglycaemia across four centres. The patterns are defined by the outcome (dependent variable) and the related independent variables included in the regression models. Each row corresponds to a specific pattern, with green indicating no missing information and red indicating missing information. Abbreviation: ID, identifier.

	Outcome	Independent variables								Per pattern: number of …	
Pattern ID	Drug-related hypoglycaemia	Age	Gender	Any insulin	Long-acting insulin	Heart failure	Diabetes mellitus type	Serum albumin decreased	Creatinine	… missing values	… encounters
1										0	11,985
2										1	5
3										1	8,210
4										2	835
5										1	52
6										2	21
7										3	1
8										2	261
9										3	11
10										4	7
11										1	797
12										2	1
13										2	905
14										3	6,394
15										2	5
16										3	1
17										4	20
18										4	3
19										4	41
20										5	563
21										3	2
Per variable: number of encounters with missing information											
Frequency	8,732	0	2	0	0	886	986	17,011	7,829		
Variable	Drug-related hypoglycaemia	Age	Gender	Any insulin	Long-acting insulin	Heart failure	Diabetes mellitus type	Serum albumin decreased	Creatinine		

Table H in S2 Supplemental tables. Meta-analysed description of the missing data pattern for the analysis (H1.c) of the outcome drug-related hypoglycaemia across six centres, which were also included in the analysis (H1.a). The patterns are defined by the outcome (dependent variable) and the related independent variables included in the regression models. Each row corresponds to a specific pattern, with green indicating no missing information and red indicating missing information. Abbreviation: ID, identifier.

Pattern ID	Outcome	Independent variables							Per pattern: number of …	
	Drug-related hypoglycaemia	Age	Gender	Any insulin	Long-acting insulin	Heart failure	Diabetes mellitus type	Serum albumin decreased	Creatinine	… missing values	… encounters
1										0	2,075
2										1	8
3										1	5,206
4										2	6,538
5										1	3
6										2	1
7										3	2
8										3	11
9										4	19
10										1	651
11										2	2
12										2	1,295
13										3	3,619
14										2	3
15										3	1
16										4	1
17										4	3
18										5	28
Per variable: number of encounters with missing information											
Frequency	5,603	0	0	0	0	61	72	16,724	10,217		
Variable	Drug-related hypoglycaemia	Age	Gender	Any insulin	Long-acting insulin	Heart failure	Diabetes mellitus type	Serum albumin decreased	Creatinine		

Table I in S2 Supplemental tables. Meta-analysed description of the study population for the analysis (B1.a) of the outcome GI bleeding across seven centres – overall as well as stratified by the outcome. Proportions for categorical variables and median values for metric variables, respectively, with 95% confidence intervals (CI) are provided. Additionally, the number of encounters (N_{Info}) building the underlying sample for the respective characteristic (in the given strata) is provided, as well as the number of encounters ($N_{Missing}$) with missing information. The analyses definitions are provided in Table 3. Further abbreviations: ALT, alanine transaminase; ASA, acetylsalicylic acid; AST, aspartate aminotransferase; GI, gastrointestinal; NSAID, non-steroidal anti-inflammatory drug; SSRI, selective serotonin reuptake inhibitor.

Characteristic	Overall			With GI bleeding			Without GI bleeding		
	$N_{Missing}$	N_{Info}	Distribution (95% CI)	$N_{Missing}$	N_{Info}	Distribution (95% CI)	$N_{Missing}$	N_{Info}	Distribution (95% CI)
GI bleeding (outcome)	5,616	330,386	1.18 (0.84, 1.66)	0	4,283	100.00 (0.00, 100.00)	0	326,103	0.00 (0.00, 100.00)
Age, in years	0	336,002	62.14 (59.90, 64.38)	0	4,283	70.46 (68.96, 71.95)	0	326,103	62.00 (59.66, 64.34)
Gender	18	335,984		0	4,283		18	326,085	
Male			51.31 (48.79, 53.83)			59.30 (57.68, 60.90)			51.17 (48.68, 53.66)
Female			48.69 (46.17, 51.21)			40.70 (39.10, 42.32)			48.83 (46.34, 51.32)
Medications	22	335,980		0	4,283		22	326,081	
NSAID			19.88 (14.23, 27.07)			5.01 (3.39, 7.34)			20.26 (14.69, 27.25)
ASA			20.81 (17.70, 24.31)			27.44 (24.24, 30.89)			20.74 (17.63, 24.24)
Bisphosphonate			0.75 (0.55, 1.02)			1.14 (0.85, 1.54)			0.74 (0.54, 1.01)
SSRI			3.89 (3.20, 4.74)			5.19 (4.37, 6.14)			3.88 (3.17, 4.75)
Diagnoses									
Liver disease	5,616	330,386	5.83 (4.17, 8.09)	0	4,283	20.70 (17.89, 23.83)	0	326,103	5.65 (4.03, 7.87)
Laboratory values									
AST increased	173,966	162,036	26.68 (23.12, 30.57)	1,260	3,023	43.99 (39.64, 48.44)	168,606	157,497	26.56 (22.88, 30.60)
ALT increased	161,956	174,046	23.04 (19.41, 27.12)	1,139	3,144	29.75 (25.55, 34.33)	156,835	169,268	23.06 (19.30, 27.31)
Serum albumin	244,546	91,456		2,037	2,246		238,308	87,795	
Normal			49.64 (36.77, 62.55)			18.32 (12.19, 26.61)			50.15 (37.25, 63.04)
Decreased			50.26 (37.31, 63.18)			81.64 (73.25, 87.83)			49.74 (36.82, 62.70)
Increased			0.06 (0.03, 0.13)			0.03 (0.00, 5.64)			0.07 (0.03, 0.14)
Haemoglobin	95,156	240,846		703	3,580		90,606	235,497	
Normal			42.99 (36.70, 49.52)			8.50 (6.20, 11.56)			43.51 (37.30, 49.94)
Decreased			55.91 (49.43, 62.20)			90.54 (86.95, 93.23)			55.40 (49.02, 61.60)
Increased			0.88 (0.54, 1.42)			0.81 (0.35, 1.86)			0.88 (0.54, 1.42)
Creatinine, in µmol/L	132,128	203,874	81.76 (78.18, 85.33)	1,177	3,106	107.70 (98.63, 116.77)	127,232	19,8871	81.63 (78.02, 85.23)

Table J in S2 Supplemental tables. Meta-analysed description of the study population for the analysis (B1.b) of the outcome GI bleeding across five centres – overall as well as stratified by the outcome. Proportions for categorical variables and median values for metric variables, respectively, with 95% confidence intervals (CI) are provided. Additionally, the number of encounters (N_{Info}) building the underlying sample for the respective characteristic (in the given strata) is provided, as well as the number of encounters ($N_{Missing}$) with missing information. The analyses definitions are provided in Table 3. Further abbreviations: ALT, alanine transaminase; ASA, acetylsalicylic acid; AST, aspartate aminotransferase; GI, gastrointestinal; NSAID, non-steroidal anti-inflammatory drug; SSRI, selective serotonin reuptake inhibitor.

Characteristic	Overall			With GI bleeding			Without GI bleeding		
	$N_{Missing}$	N_{Info}	Distribution (95% CI)	$N_{Missing}$	N_{Info}	Distribution (95% CI)	$N_{Missing}$	N_{Info}	Distribution (95% CI)
GI bleeding (outcome)	5,535	237,220	1.26 (0.79, 1.99)	0	3,349	100.00 (0.00, 100.00)	0	233,871	0.00 (0.00, 100.00)
Age, in years	0	242,755	63.20 (60.62, 65.79)	0	3,349	71.15 (69.48, 72.83)	0	233,871	63.20 (60.62, 65.79)
Gender	17	242,738		0	3,349		17	233,854	
Male			52.29 (49.53, 55.03)			59.65 (57.79, 61.49)			52.12 (49.42, 54.82)
Female			47.71 (44.97, 50.47)			40.35 (38.51, 42.21)			47.88 (45.18, 50.58)
Medications	0	242,755		0	3,349		0	233,871	
NSAID			18.15 (11.74, 26.99)			4.94 (2.87, 8.38)			18.57 (12.25, 27.15)
ASA			23.34 (20.92, 25.95)			29.69 (26.88, 32.66)			23.27 (20.85, 25.89)
Bisphosphonate			0.85 (0.67, 1.08)			1.24 (0.87, 1.75)			0.85 (0.67, 1.07)
SSRI			4.15 (3.25, 5.28)			5.20 (4.22, 6.39)			4.15 (3.23, 5.30)
Diagnoses									
Liver disease	5,535	237,220	6.22 (4.05, 9.45)	0	3,349	21.42 (17.74, 25.62)	0	233,871	6.03 (3.92, 9.18)
Laboratory values									
AST increased	116,914	125,841	26.05 (21.35, 31.37)	954	2,395	42.99 (38.05, 48.07)	111,913	121,958	26.00 (21.11, 31.57)
ALT increased	105,643	137,112	22.35 (17.64, 27.90)	891	2,458	29.02 (23.49, 35.23)	100,822	133,049	22.44 (17.53, 28.26)
Serum albumin	166,895	75,860		1,470	1,879		161,291	72,580	
Normal			57.93 (48.88, 66.48)			23.54 (17.65, 30.66)			58.39 (49.27, 66.96)
Decreased			41.94 (33.35, 51.05)			76.37 (69.11, 82.37)			41.49 (32.87, 50.66)
Increased			0.09 (0.04, 0.19)			0.07 (0.00, 3.76)			0.09 (0.04, 0.20)
Haemoglobin	66,767	175,988		648	2,701		62,310	171,561	
Normal			41.23 (33.28, 49.66)			7.46 (5.29, 10.41)			41.76 (33.91, 50.05)
Decreased			57.84 (49.49, 65.76)			91.86 (88.61, 94.24)			57.31 (49.11, 65.13)
Increased			0.75 (0.43, 1.29)			0.51 (0.15, 1.77)			0.75 (0.43, 1.30)
Creatinine, in µmol/L	70,152	172,603	83.36 (79.93, 86.79)	671	2,678	110.45 (99.91, 120.98)	65,837	168,034	83.18 (79.61, 86.75)

Table K in S2 Supplemental tables. Meta-analysed description of the study population for the analysis (B1.c) of the outcome GI bleeding across the seven centres, which were also included in the analysis (B1.a). Proportions for categorical variables and median values for metric variables, respectively, with 95% confidence intervals (CI) are provided. Additionally, the number of encounters (N_{Info}) building the underlying sample for the respective characteristic is provided, as well as the number of encounters ($N_{Missing}$) with missing information. The analyses definitions are provided in Table 3. Within the characteristics derived from diagnoses, the larger number of encounters with missing information for GI bleeding can be attributed to the exclusion of encounters due to coded GI bleeding on the day of admission. Further abbreviations: ALT, alanine transaminase; ASA, acetylsalicylic acid; AST, aspartate aminotransferase; GI, gastrointestinal; NSAID, non-steroidal anti-inflammatory drug; SSRI, selective serotonin reuptake inhibitor.

Characteristic	Overall		
	$N_{Missing}$	N_{Info}	Distribution (95% CI)
GI bleeding (outcome)	7,941	328,061	0.02 (0.01, 0.06)
Age, in years	0	336,002	62.14 (59.90, 64.38)
Gender	18	335,984	
Male			51.31 (48.79, 53.83)
Female			48.69 (46.17, 51.21)
Medications	22	335,980	
NSAID			6.97 (3.69, 12.75)
ASA			3.73 (1.72, 7.92)
Bisphosphonate			0.10 (0.03, 0.29)
SSRI			0.58 (0.23, 1.44)
Diagnoses			
Liver disease	5,616	330,386	0.79 (0.11, 5.38)
Laboratory values			
AST increased	239,681	96,321	17.23 (13.94, 21.11)
ALT increased	232,028	103,974	15.80 (13.42, 18.52)
Serum albumin	289,468	46,534	
Normal			76.40 (58.95, 87.95)
Decreased			23.39 (11.86, 40.91)
Increased			0.10 (0.04, 0.21)
Haemoglobin	188,016	147,986	
Normal			59.75 (54.27, 65.00)
Decreased			39.14 (33.99, 44.55)
Increased			0.84 (0.48, 1.45)
Creatinine, in µmol/L	215,289	120,713	79.49 (75.53, 83.46)

Table L in S2 Supplemental tables. Meta-analysed description of the study population for the analysis (H1.a) of the outcome drug-related hypoglycaemia across six centres – overall as well as stratified by the outcome. Proportions for categorical variables and median values for metric variables, respectively, with 95% confidence intervals (CI) are provided. Additionally, the number of encounters (N_{Info}) building the underlying sample for the respective characteristic (in the given strata) is provided, as well as the number of encounters ($N_{Missing}$) with missing information. The analyses definitions are provided in Table 3. Within the characteristics derived from diagnoses, the larger number of encounters with missing information for diabetes mellitus (DM) can be attributed to the exclusion of encounters with documented type 1 DM and type 2 DM (so-called “double diabetes”).

Characteristic	Overall			With drug-related hypoglycaemia			Without drug-related hypoglycaemia		
	$N_{Missing}$	N_{Info}	Distribution (95% CI)	$N_{Missing}$	N_{Info}	Distribution (95% CI)	$N_{Missing}$	N_{Info}	Distribution (95% CI)
Drug-related hypoglycaemia (outcome)	11,110	32,991	2.93 (2.15, 3.99)	0	1,027	100.00 (0.00, 100.00)	0	31,964	0.00 (0.00, 100.00)
Age, in years	0	44,101	70.17 (68.99, 71.34)	0	1,027	72.21 (71.05, 73.36)	0	31,964	70.68 (69.86, 71.49)
Gender	2	44,099		0	1,027		0	31,964	
Male			62.51 (60.74, 64.25)			57.49 (53.49, 61.39)			62.50 (60.46, 64.50)
Female			37.49 (35.75, 39.26)			42.51 (38.61, 46.51)			37.50 (35.50, 39.54)
Medications	0	44,101		0	1,027		0	31,964	
Antihyperglycaemic drug			100.00 (0.00, 100.00)			100.00 (0.00, 100.00)			100.00 (0.00, 100.00)
Any insulin			57.86 (52.02, 63.49)			86.77 (82.20, 90.31)			59.36 (52.30, 66.06)
Long-acting insulin			25.66 (20.68, 31.37)			46.30 (40.33, 52.37)			25.48 (20.13, 31.68)
Diagnoses									
Heart failure	953	43,148	17.59 (13.22, 23.02)	6	1,021	23.53 (18.85, 28.97)	308	31,656	18.00 (13.82, 23.10)
Diabetes mellitus (DM)	1,071	43,030		18	1,009		386	31,578	
No DM			25.32 (18.75, 33.25)			13.31 (8.29, 20.67)			23.12 (16.15, 31.94)
DM type 1			2.39 (1.90, 2.99)			9.32 (7.67, 11.27)			2.09 (1.60, 2.73)
DM type 2			68.90 (61.22, 75.66)			69.32 (64.00, 74.17)			71.32 (62.94, 78.46)
DM other type			2.86 (2.28, 3.58)			6.08 (3.87, 9.43)			2.74 (2.08, 3.60)
Laboratory values									
Serum albumin	28,726	15,375		376	651		18,160	13,804	
Normal			43.65 (28.46, 60.13)			21.51 (13.88, 31.78)			44.56 (29.53, 60.64)
Decreased			56.25 (39.72, 71.50)			78.48 (67.99, 86.23)			55.34 (39.21, 70.43)
Increased			0.08 (0.03, 0.26)			0.15 (0.02, 1.08)			0.09 (0.03, 0.26)
Creatinine, in µmol/L	10,874	33,227	98.78 (93.02, 104.53)	23	1,004	133.66 (114.29, 153.04)	2,397	29,567	98.77 (92.99, 104.55)

Table M in S2 Supplemental tables. Meta-analysed description of the study population for the analysis (H1.b) of the outcome drug-related hypoglycaemia across four centres – overall as well as stratified by the outcome. Proportions for categorical variables and median values for metric variables, respectively, with 95% confidence intervals (CI) are provided. Additionally, the number of encounters (N_{Info}) building the underlying sample for the respective characteristic (in the given strata) is provided, as well as the number of encounters ($N_{Missing}$) with missing information. The analyses definitions are provided in Table 3. Within the characteristics derived from diagnoses, the larger number of encounters with missing information for diabetes mellitus (DM) can be attributed to the exclusion of encounters with documented type 1 DM and type 2 DM (so-called "double diabetes").

Characteristic	Overall			With drug-related hypoglycaemia			Without drug-related hypoglycaemia		
	$N_{Missing}$	N_{Info}	Distribution (95% CI)	$N_{Missing}$	N_{Info}	Distribution (95% CI)	$N_{Missing}$	N_{Info}	Distribution (95% CI)
Drug-related hypoglycaemia (outcome)	8,732	21,388	3.64 (2.89, 4.56)	0	785	100.00 (0.00, 100.00)	0	20,603	0.00 (0.00, 100.00)
Age, in years	0	30,120	70.75 (69.51, 71.98)	0	785	71.86 (70.62, 73.10)	0	20,603	71.00 (70.21, 71.80)
Gender	2	30,118		0	785		0	20,603	
Male			63.55 (62.42, 64.67)			59.62 (56.14, 63.00)			63.82 (62.33, 65.28)
Female			36.45 (35.33, 37.58)			40.38 (37.00, 43.86)			36.18 (34.72, 37.67)
Medications	0	30,120		0	785		0	20,603	
Antihyperglycaemic drug			100.00 (0.00, 100.00)			100.00 (0.00, 100.00)			100.00 (0.00, 100.00)
Any insulin			55.24 (49.84, 60.52)			87.58 (81.18, 92.02)			55.50 (50.23, 60.65)
Long-acting insulin			28.24 (22.85, 34.34)			48.40 (40.77, 56.09)			28.31 (22.68, 34.71)
Diagnoses									
Heart failure	886	29,234	20.79 (15.65, 27.08)	2	783	24.00 (17.98, 31.28)	277	20,326	20.76 (15.49, 27.26)
Diabetes mellitus (DM)	986	29,134		13	772		340	20,263	
No DM			21.60 (18.69, 24.82)			11.24 (5.78, 20.73)			18.71 (15.37, 22.57)
DM type 1			2.36 (2.19, 2.54)			9.20 (7.35, 11.45)			2.21 (2.02, 2.42)
DM type 2			72.99 (69.37, 76.34)			70.02 (62.53, 76.58)			76.01 (72.08, 79.54)
DM other type			2.89 (2.13, 3.89)			7.87 (5.86, 10.51)			2.86 (2.07, 3.95)
Laboratory values									
Serum albumin	17,011	13,109		218	567		8,867	11,736	
Normal			48.94 (37.84, 60.14)			24.50 (17.41, 33.30)			49.52 (39.05, 60.03)
Decreased			50.93 (39.63, 62.14)			75.38 (66.34, 82.63)			50.35 (39.75, 60.92)
Increased			0.10 (0.03, 0.33)			0.18 (0.02, 1.24)			0.11 (0.04, 0.33)
Creatinine, in µmol/L	7,829	22,291	101.15 (94.96, 107.34)	8	777	139.70 (117.53, 161.88)	840	19,763	100.93 (94.67, 107.18)

Table N in S2 Supplemental tables. Meta-analysed description of the study population for the analysis (H1.c) of the outcome drug-related hypoglycaemia across the six centres, which were also included in the analysis (H1.a). Proportions for categorical variables and median values for metric variables, respectively, with 95% confidence intervals (CI) are provided. Additionally, the number of encounters (N_{Info}) building the underlying sample for the respective characteristic is provided, as well as the number of encounters ($N_{Missing}$) with missing information. The analyses definitions are provided in Table 3. Within the characteristics derived from diagnoses, the larger number of encounters with missing information for diabetes mellitus (DM) can be attributed to the exclusion of encounters with documented type 1 DM and type 2 DM (so-called "double diabetes").

Characteristic	Overall		
	$N_{Missing}$	N_{Info}	Distribution (95% CI)
Drug-related hypoglycaemia (outcome)	5,603	13,863	2.19 (1.38, 3.45)
Age, in years	0	19,466	70.19 (68.80, 71.57)
Gender	0	19,466	
Male			63.00 (60.83, 65.12)
Female			37.00 (34.88, 39.17)
Medications	0	19,466	
Antihyperglycaemic drug			100.00 (0.00, 100.00)
Any insulin			55.37 (48.09, 62.43)
Long-acting insulin			25.40 (20.05, 31.62)
Diagnoses			
Heart failure	61	19,405	1.46 (0.26, 7.80)
Diabetes mellitus (DM)	72	19,394	
No DM			92.02 (44.31, 99.40)
DM type 1			0.46 (0.13, 1.60)
DM type 2			7.08 (0.57, 50.33)
DM other type			0.51 (0.14, 1.87)
Laboratory values			
Serum albumin	16,724	2,742	
Normal			75.15 (56.08, 87.75)
Decreased			24.75 (12.16, 43.85)
Increased			0.11 (0.04, 0.34)
Creatinine, in µmol/L	10,217	9,249	91.57 (84.99, 98.15)

Table O in S2 Supplemental tables. Meta-analysed results from the regression modelling for the analysis (B1.a) of the outcome GI bleeding across seven centres. For each model, a model summary of the meta-analysed regression models as well as summarised information of the local regression results are provided. The analyses definitions are provided in Table 3. Note that the odds ratio for metric covariates must be read as an increase in odds per 1 unit increase of the covariate. Further abbreviations: AIC, Akaike information criterion; ALT, alanine transaminase; ASA, acetylsalicylic acid; AST, aspartate aminotransferase; ROC AUC, area under the receiver operating characteristic curve; BIC, Bayes information criterion; CI, confidence interval; GI, gastrointestinal; LR, likelihood-ratio; NSAID, non-steroidal anti-inflammatory drug; N, number of encounters included in the respective model; OR, odds ratio; Q1/Q3, first/third quartile; SSRI, selective serotonin reuptake inhibitor; VIF, variance inflation factor.

Model	Variable	Model summary					Model evaluation: Descriptive summary of local results		
		N	OR (95% CI)	AIC	BIC	I^2	Centres with LR test p-value <0.05 [n (%)]	ROC AUC [median (Q1, Q3)]	VIF [median (Q1, Q3)]
Univariable models									
1	Age, in 10 years	330,386	1.31 (1.28, 1.34)	-38.2	-35.7	97.3%	7 (100.00%)	0.63 (0.62, 0.64)	-
2	Male gender	330,368	1.40 (1.24, 1.59)	4.2	6.6	97.6%	6 (85.71%)	0.54 (0.53, 0.55)	-
3	NSAID	330,364	0.21 (0.15, 0.30)	24.0	26.4	97.1%	7 (100.00%)	0.58 (0.56, 0.60)	-
4	ASA	330,364	1.43 (1.29, 1.59)	4.0	6.5	97.6%	7 (100.00%)	0.53 (0.53, 0.54)	-
5	Bisphosphonate	330,364	1.59 (1.04, 2.43)	27.7	30.1	97.7%	1 (14.29%)	0.50 (0.50, 0.50)	-
6	SSRI	330,364	1.34 (1.04, 1.72)	22.4	24.8	97.7%	3 (42.86%)	0.51 (0.50, 0.51)	-
7	Liver disease	330,386	4.32 (3.26, 5.73)	15.4	17.8	97.3%	7 (100.00%)	0.57 (0.57, 0.58)	-
8	AST increased	160,520	2.19 (1.80, 2.66)	13.1	15.5	95.2%	7 (100.00%)	0.58 (0.56, 0.60)	-
9	ALT increased	172,412	1.41 (1.21, 1.64)	16.1	18.5	96.8%	4 (57.14%)	0.53 (0.52, 0.55)	-
10	Serum albumin decreased	90,041	4.72 (3.83, 5.81)	11.2	13.6	86.9%	7 (100.00%)	0.66 (0.64, 0.68)	-
11	Haemoglobin decreased	239,077	7.61 (5.62, 10.30)	21.5	23.9	96.0%	7 (100.00%)	0.66 (0.65, 0.70)	-
12	Creatinine, in mmol/L	201,977	6.12 (2.65, 14.12)	-46.4	-44.0	97.2%	7 (100.00%)	0.67 (0.63, 0.68)	-
Base model (multivariable)									
13		330,346	-	12.7	95.1	87.3%	7 (100.00%)	0.70 (0.69, 0.73)	-
	Age, in 10 years	-	1.27 (1.24, 1.30)	-	-		-	-	1.07 (1.06, 1.08)
	Male gender	-	1.21 (1.10, 1.34)	-	-		-	-	1.02 (1.02, 1.03)
	NSAID	-	0.30 (0.22, 0.42)	-	-		-	-	1.02 (1.02, 1.04)
	ASA	-	1.08 (0.98, 1.18)	-	-		-	-	1.06 (1.05, 1.06)
	Bisphosphonate	-	1.19 (0.76, 1.84)	-	-		-	-	1.01 (1.00, 1.01)
	SSRI	-	1.32 (1.06, 1.64)	-	-		-	-	1.00 (1.00, 1.01)
	Liver disease	-	4.13 (3.24, 5.26)	-	-		-	-	1.02 (1.02, 1.02)

Table P in S2 Supplemental tables. Meta-analysed results from the regression modelling for the analysis (B1.b) of the outcome GI bleeding across five centres. For each model, a model summary of the meta-analysed regression models as well as summarised information of the local regression results are provided. The analyses definitions are provided in Table 3. Note that the odds ratio for metric covariates must be read as an increase in odds per 1 unit increase of the covariate. Further abbreviations: AIC, Akaike information criterion; ALT, alanine transaminase; ASA, acetylsalicylic acid; AST, aspartate aminotransferase; ROC AUC, area under the receiver operating characteristic curve; BIC, Bayes information criterion; CI, confidence interval; GI, gastrointestinal; LR, likelihood-ratio; NSAID, non-steroidal anti-inflammatory drug; N, number of encounters included in the respective model; OR, odds ratio; Q1/Q3, first/third quartile; SSRI, selective serotonin reuptake inhibitor; VIF, variance inflation factor.

Model ID	Variable	Model summary					Model evaluation: Descriptive summary of local results		
		N	OR (95% CI)	AIC	BIC	I^2	Centres with LR test p-value <0.05 [n (%)]	ROC AUC [median (Q1, Q3)]	VIF [median (Q1, Q3)]
Univariable models									
1	Age, in 10 years	237,220	1.31 (1.26, 1.35)	-21.6	-21.2	97.7%	5 (100.00%)	0.62 (0.61, 0.63)	-
2	Male gender	237,203	1.39 (1.16, 1.67)	7.6	8.0	97.8%	4 (80.00%)	0.53 (0.53, 0.54)	-
3	NSAID	237,220	0.23 (0.15, 0.37)	21.5	21.9	97.3%	5 (100.00%)	0.57 (0.54, 0.58)	-
4	ASA	237,220	1.38 (1.23, 1.55)	6.6	7.0	97.9%	5 (100.00%)	0.53 (0.52, 0.54)	-
5	Bisphosphonate	237,220	1.59 (0.93, 2.71)	22.2	22.6	97.9%	1 (20.00%)	0.50 (0.50, 0.50)	-
6	SSRI	237,220	1.25 (0.95, 1.64)	18.6	19.0	98.0%	2 (40.00%)	0.51 (0.50, 0.51)	-
7	Liver disease	237,220	4.25 (2.84, 6.35)	12.0	12.4	97.5%	5 (100.00%)	0.57 (0.56, 0.59)	-
8	AST increased	124,353	2.17 (1.72, 2.73)	12.1	12.5	96.4%	5 (100.00%)	0.58 (0.57, 0.60)	-
9	ALT increased	135,507	1.41 (1.17, 1.70)	15.7	16.1	97.8%	3 (60.00%)	0.53 (0.52, 0.54)	-
10	Serum albumin decreased	74,459	4.57 (3.53, 5.92)	7.5	7.9	81.1%	5 (100.00%)	0.68 (0.66, 0.69)	-
11	Haemoglobin decreased	174,262	8.22 (6.20, 10.91)	17.0	17.4	96.9%	5 (100.00%)	0.66 (0.65, 0.70)	-
12	Creatinine, in mmol/L	170,712	5.81 (2.75, 12.26)	-28.9	-28.5	97.7%	5 (100.00%)	0.67 (0.65, 0.69)	-
Base model (multivariable)									
13		237,203	-	36.1	100.6	89.2%	5 (100.00%)	0.70 (0.68, 0.70)	-
	Age, in 10 years	-	1.27 (1.24, 1.31)	-	-		-	-	1.06 (1.06, 1.07)
	Male gender	-	1.20 (1.05, 1.38)	-	-		-	-	1.02 (1.02, 1.02)
	NSAID	-	0.32 (0.21, 0.50)	-	-		-	-	1.02 (1.01, 1.03)
	ASA	-	1.08 (0.96, 1.20)	-	-		-	-	1.06 (1.05, 1.06)
	Bisphosphonate	-	1.21 (0.72, 2.03)	-	-		-	-	1.01 (1.00, 1.01)
	SSRI	-	1.28 (0.97, 1.67)	-	-		-	-	1.00 (1.00, 1.00)
	Liver disease	-	4.16 (2.91, 5.94)	-	-		-	-	1.02 (1.02, 1.02)

Extended model (multivariable)									
14		61,317	-	84.3	287.2	41.9%	5 (100.00%)	0.75 (0.72, 0.76)	-
	Age, in 10 years	-	1.10 (1.06, 1.15)	-	-		-	-	1.09 (1.08, 1.10)
	Male gender	-	1.04 (0.90, 1.20)	-	-		-	-	1.05 (1.05, 1.05)
	NSAID	-	0.37 (0.23, 0.62)	-	-		-	-	1.02 (1.02, 1.03)
	ASA	-	0.85 (0.76, 0.96)	-	-		-	-	1.06 (1.05, 1.08)
	Bisphosphonate	-	1.14 (0.66, 1.97)	-	-		-	-	1.01 (1.00, 1.01)
	SSRI	-	1.22 (0.97, 1.53)	-	-		-	-	1.01 (1.01, 1.01)
	Liver disease	-	2.10 (1.79, 2.46)	-	-		-	-	1.11 (1.11, 1.13)
	AST increased	-	1.17 (0.95, 1.43)	-	-		-	-	1.63 (1.63, 1.77)
	ALT increased	-	0.89 (0.69, 1.14)	-	-		-	-	1.56 (1.52, 1.61)
	Serum albumin decreased	-	2.50 (2.07, 3.02)	-	-		-	-	1.16 (1.16, 1.17)
	Haemoglobin decreased	-	3.57 (2.23, 5.71)	-	-		-	-	1.10 (1.09, 1.12)
	Creatinine, in mmol/L	-	2.22 (1.25, 3.97)	-	-		-	-	1.03 (1.03, 1.03)

Table Q in S2 Supplemental tables. Meta-analysed results from the regression modelling for the analysis (H1.a) of the outcome drug-related hypoglycaemia across six centres. For each model, a model summary of the meta-analysed regression models as well as summarised information of the local regression results are provided. The analyses definitions are provided in Table 3. Note that the odds ratio for metric covariates must be read as an increase in odds per 1 unit increase of the covariate. Further abbreviations: AIC, Akaike information criterion; ROC AUC, area under the receiver operating characteristic curve; BIC, Bayes information criterion; CI, confidence interval; DM, diabetes mellitus; LR, likelihood-ratio; N, number of encounters included in the respective model; OR, odds ratio; Q1/Q3, first/third quartile; ref., reference; VIF, variance inflation factor

Model ID	Variable	Model summary					Model evaluation: Descriptive summary of local results		
		N	OR (95% CI)	AIC	BIC	I^2	Centres with LR test p-value <0.05 [n (%)]	ROC AUC [median (Q1, Q3)]	VIF [median (Q1, Q3)]
Univariable models									
1	Age, in 10 years	32,991	1.03 (0.97, 1.10)	-19.1	-17.6	92.2%	1 (16.67%)	0.53 (0.51, 0.54)	-
2	Male gender	32,991	0.81 (0.70, 0.92)	13.2	14.7	92.3%	2 (33.33%)	0.53 (0.51, 0.54)	-
3	Any insulin	32,991	4.17 (2.68, 6.50)	19.7	21.2	93.6%	6 (100.00%)	0.65 (0.64, 0.66)	-
4	Long-acting insulin	32,991	2.55 (2.07, 3.14)	16.8	18.3	93.0%	6 (100.00%)	0.59 (0.58, 0.62)	-
5	Heart failure	32,677	1.37 (1.10, 1.71)	16.0	17.5	91.9%	1 (16.67%)	0.52 (0.51, 0.52)	-
6	Diabetes mellitus (DM) *	32,587	-	47.0	61.0	87.5%	6 (100.00%)	0.58 (0.57, 0.60)	
	DM type 1	-	7.95 (4.45, 14.21)	-	-		-	-	-
	DM type 2	-	1.71 (1.09, 2.68)	-	-		-	-	-
	DM other type	-	4.25 (2.84, 6.38)	-	-		-	-	-
7	Serum albumin decreased	14,455	3.15 (2.59, 3.83)	28.5	30.0	82.0%	6 (100.00%)	0.62 (0.60, 0.64)	-
8	Creatinine, in mmol/L	30,571	6.19 (3.51, 10.91)	-43.9	-42.4	90.4%	5 (83.33%)	0.62 (0.61, 0.64)	-
Base model (multivariable)									
9		32,587	-	77.0	174.5	77.3%	6 (100.00%)	0.72 (0.72, 0.73)	-
	Age, in 10 years	-	1.11 (1.05, 1.18)	-	-		-	-	1.19 (1.17, 1.23)
	Male gender	-	0.81 (0.67, 0.97)	-	-		-	-	1.01 (1.01, 1.01)
	Any insulin	-	3.86 (2.61, 5.72)	-	-		-	-	1.22 (1.20, 1.34)
	Long-acting insulin	-	1.18 (0.86, 1.62)	-	-		-	-	1.36 (1.26, 1.40)
	Heart failure	-	1.37 (1.15, 1.62)	-	-		-	-	1.05 (1.04, 1.07)
	Diabetes mellitus (DM) *	-	-	-	-		-	-	1.31 (1.26, 1.37)
	DM type 1	-	5.97 (3.39, 10.51)	-	-		-	-	-
	DM type 2	-	1.60 (1.03, 2.47)	-	-		-	-	-
	DM other type	-	3.04 (1.93, 4.79)	-	-		-	-	-

*The reference category is “no DM”.

Table R in S2 Supplemental tables. Meta-analysed results from the regression modelling for the analysis (H1.b) of the outcome drug-related hypoglycaemia across four centres. For each model, a model summary of the meta-analysed regression models as well as summarised information of the local regression results are provided. The analyses definitions are provided in Table 3. Note that the odds ratio for metric covariates must be read as an increase in odds per 1 unit increase of the covariate. Further abbreviations: AIC, Akaike information criterion; ROC AUC, area under the receiver operating characteristic curve; BIC, Bayes information criterion; CI, confidence interval; DM, diabetes mellitus; LR, likelihood-ratio; N, number of encounters included in the respective model; OR, odds ratio; Q1/Q3, first/third quartile; ref., reference; VIF, variance inflation factor

Model ID	Variable	Model summary					Model evaluation: Descriptive summary of local results		
		N	OR (95% CI)	AIC	BIC	I^2	Centres with LR test p-value <0.05 [n (%)]	ROC AUC [median (Q1, Q3)]	VIF [median (Q1, Q3)]
Univariable models									
1	Age, in 10 years	21,388	0.99 (0.93, 1.05)	-11.8	-12.8	88.4%	0 (0.00%)	0.51 (0.50, 0.53)	-
2	Male gender	21,388	0.84 (0.71, 1.00)	8.5	7.5	88.9%	2 (50.00%)	0.52 (0.51, 0.54)	-
3	Any insulin	21,388	5.58 (3.93, 7.93)	8.0	7.0	83.6%	4 (100.00%)	0.66 (0.65, 0.66)	-
4	Long-acting insulin	21,388	2.38 (1.97, 2.87)	10.6	9.5	91.9%	4 (100.00%)	0.59 (0.58, 0.61)	-
5	Heart failure	21,109	1.18 (0.98, 1.41)	6.7	5.7	87.4%	0 (0.00%)	0.52 (0.51, 0.52)	-
6	Diabetes mellitus (DM) *	21,035	-	28.4	35.2	81.0%	4 (100.00%)	0.58 (0.56, 0.59)	
	DM type 1	-	7.17 (3.63, 14.18)	-	-	-	-	-	-
	DM type 2	-	1.55 (0.89, 2.71)	-	-	-	-	-	-
	DM other type	-	4.50 (2.49, 8.15)	-	-	-	-	-	-
7	Serum albumin decreased	12,303	2.99 (2.41, 3.70)	9.4	8.3	86.3%	4 (100.00%)	0.62 (0.61, 0.63)	-
8	Creatinine, in mmol/L	20,540	5.56 (2.52, 12.26)	-23.3	-24.3	89.3%	3 (75.00%)	0.63 (0.60, 0.65)	-
Base model (multivariable)									
9		21,035	-	73.0	143.0	62.8%	4 (100.00%)	0.72 (0.72, 0.73)	-
	Age, in 10 years	-	1.10 (1.02, 1.18)	-	-	-	-	-	1.21 (1.19, 1.30)
	Male gender	-	0.88 (0.70, 1.10)	-	-	-	-	-	1.01 (1.01, 1.02)
	Any insulin	-	4.88 (3.35, 7.11)	-	-	-	-	-	1.28 (1.16, 1.39)
	Long-acting insulin	-	1.10 (0.76, 1.59)	-	-	-	-	-	1.31 (1.19, 1.39)
	Heart failure	-	1.33 (1.10, 1.62)	-	-	-	-	-	1.05 (1.05, 1.07)
	Diabetes mellitus (DM) *	-	-	-	-	-	-	-	1.27 (1.24, 1.35)
	DM type 1	-	5.07 (2.38, 10.79)	-	-	-	-	-	-
	DM type 2	-	1.38 (0.78, 2.46)	-	-	-	-	-	-
	DM other type	-	3.35 (1.70, 6.62)	-	-	-	-	-	-

Extended model (multivariable)									
10		11,985	-	87.4	202.6	49.9%	4 (100.00%)	0.76 (0.75, 0.76)	-
	Age, in 10 years	-	1.03 (0.95, 1.11)	-	-	-	-	-	1.23 (1.19, 1.32)
	Male gender	-	0.86 (0.66, 1.14)	-	-	-	-	-	1.02 (1.02, 1.03)
	Any insulin	-	3.32 (2.05, 5.39)	-	-	-	-	-	1.24 (1.13, 1.37)
	Long-acting insulin	-	1.13 (0.69, 1.84)	-	-	-	-	-	1.31 (1.21, 1.39)
	Heart failure	-	1.11 (0.88, 1.42)	-	-	-	-	-	1.08 (1.08, 1.10)
	Diabetes mellitus (DM) *	-	-	-	-	-	-	-	1.43 (1.39, 1.46)
	DM type 1	-	5.55 (2.47, 12.45)	-	-	-	-	-	-
	DM type 2	-	1.57 (0.87, 2.85)	-	-	-	-	-	-
	DM other type	-	3.46 (1.76, 6.80)	-	-	-	-	-	-
	Serum albumin decreased	-	2.48 (1.89, 3.25)	-	-	-	-	-	1.07 (1.06, 1.09)
	Creatinine, in mmol/L	-	2.74 (1.33, 5.67)	-	-	-	-	-	1.06 (1.05, 1.08)

*The reference category is "no DM".
