# Supplementary material for: Challenges in detecting and predicting adverse drug events via distributed analysis of electronic health record data from German university hospitals
Source: PLOS Digit Health. 2025 Jun 26;4(6):e0000892. doi: 10.1371/journal.pdig.0000892 (PMC12200832; doi:10.1371/journal.pdig.0000892)
Supplement: S3 File — (PDF) [file pdig.0000892.s003.pdf]

Supporting information file S3: Supplemental figures

Challenges in detecting and predicting adverse drug events via distributed analysis of electronic health record data from German university hospitals

Anna Maria Wermund[1], Torsten Thalheim[2,3,4], André Medek[5], Florian Schmidt[3], Thomas Peschel[3], Alexander Strübing[3], Daniel Neumann[3], André Scherag[6], Markus Loeffler[3], Miriam Kesselmeier[6,¶] and Ulrich Jaehde[1,¶*] for the POLAR_MI Consortium[+]

[+] The membership list of POLAR_MI is provided in Supporting information file S1 (“Membership list of POLAR_MI”)

[1] Department of Clinical Pharmacy, Institute of Pharmacy, University of Bonn, Bonn, Germany.
[2] Interdisciplinary Centre for Bioinformatics, Leipzig University, Leipzig, Germany
[3] Institute for Medical Informatics, Statistics and Epidemiology (IMISE), Leipzig University, Leipzig, Germany
[4] Deutsches Biomasseforschungszentrum gGmbH, Torgauer Str. 116, 04347 Leipzig, Germany
[5] Medical & Scientific Technology Development & Coordination (MWTek), University Hospital Bonn, 53127 Bonn, Germany
[6] Institute of Medical Statistics, Computer and Data Sciences (IMSID), Jena University Hospital – Friedrich Schiller University Jena, Jena, Germany

[¶] Equal contribution

* u.jaehde@uni-bonn.de

List of supplemental figures

Fig A in S3 Supplemental figures. Number of included and excluded encounters (together with the exclusion reasons) for the outcome GI bleeding for the analyses (B1.a) and (B1.b) 3

Fig B in S3 Supplemental figures. Number of included and excluded encounters (together with the exclusion reasons) for the outcome drug-related hypoglycaemia for the analyses (H1.a) and (H1.b) 4

The initial Sankey diagrams in Fig A and Fig B were generated with the *Sankey Diagram Generator* by Dénes Csala, based on the *Sankey plugin for D3* by Mike Bostock (https://sankey.csaladen.es; 2014), and subsequently adapted on 2025/01/16.

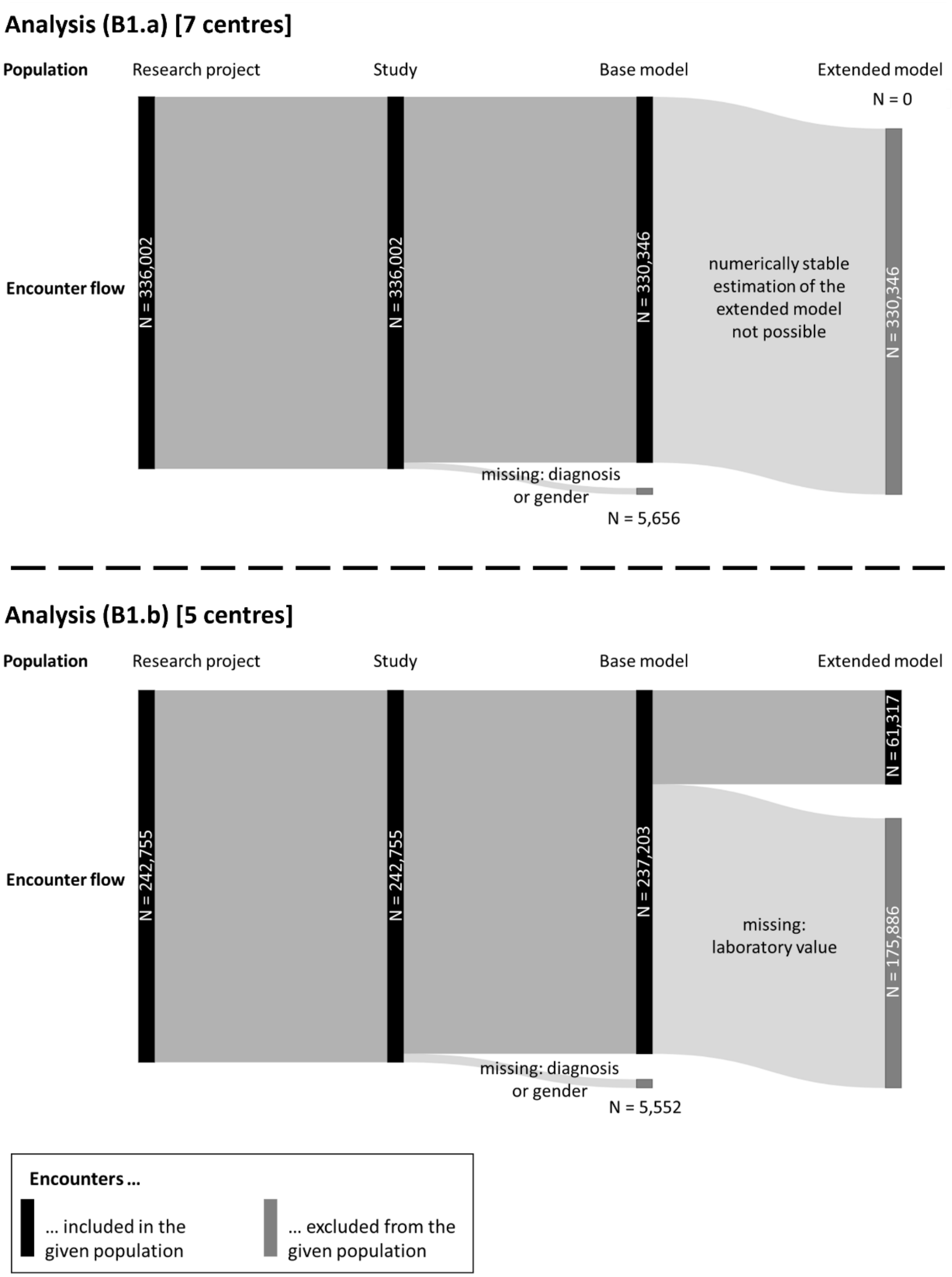

Fig A in S3 Supplemental figures. Number of included and excluded encounters (together with the exclusion reasons) for the outcome GI bleeding for the analyses (B1.a) and (B1.b). These numbers (N) are provided for all populations from the research project population to the extended model population. Please note, that there was no additional inclusion criterion for the outcome GI bleeding, so that the related study population was identical to the overall population of our research project. Furthermore, the extended model population was empty for the analysis (B1.a). The definitions of the analyses are provided in Table 3.

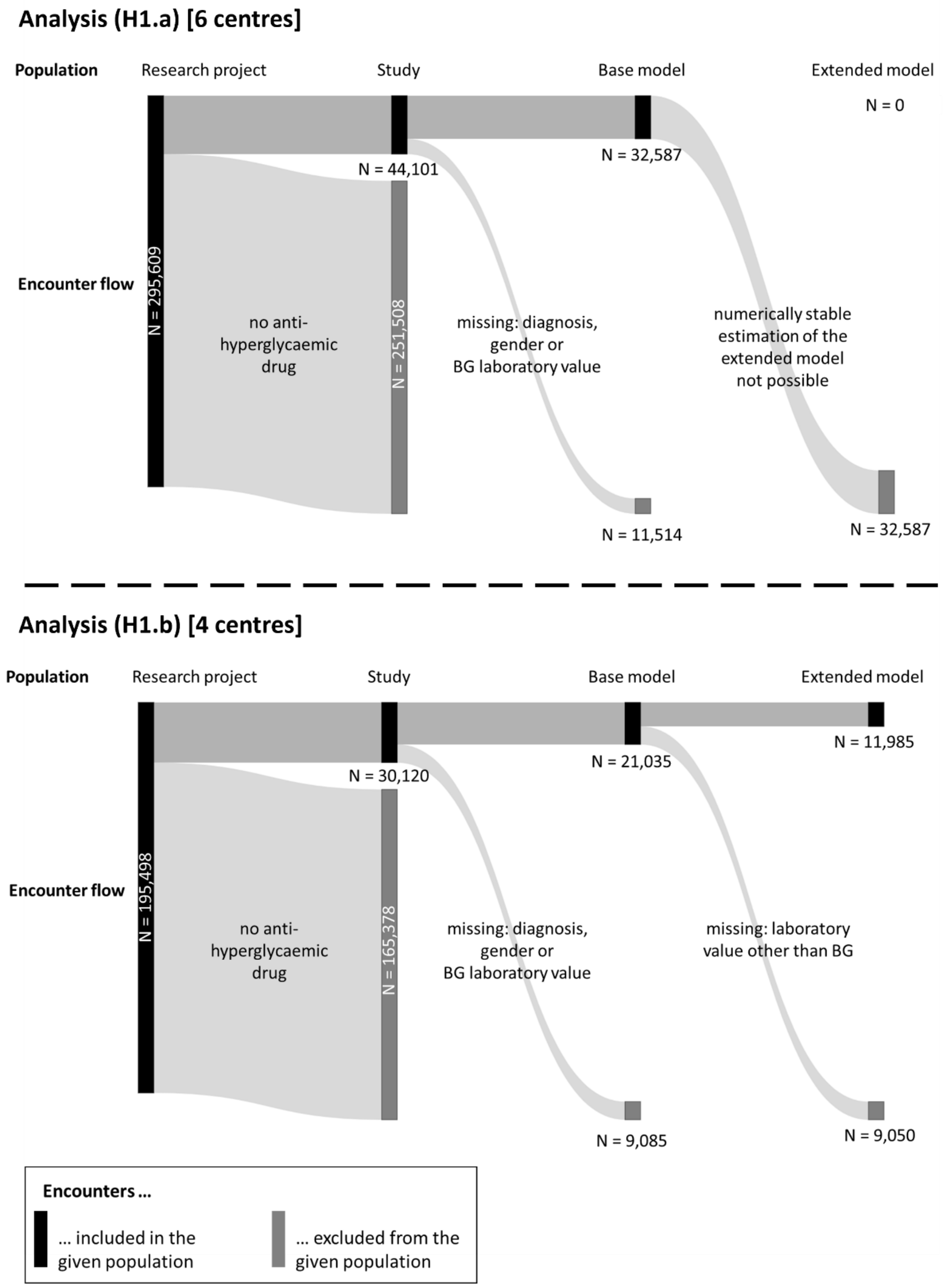

Fig B in S3 Supplemental figures. Number of included and excluded encounters (together with the exclusion reasons) for the outcome drug-related hypoglycaemia for the analyses (H1.a) and (H1.b). These numbers (N) are provided for all populations from the research project population to the extended model population. Please note, that the extended model population was empty for the analysis (H1.a). The definitions of the analyses are provided in Table 3. Further abbreviation: BG, blood glucose.
